# Aging impairs human bone marrow function and cardiac repair following myocardial infarction in a humanized chimeric mouse

**DOI:** 10.1111/acel.13494

**Published:** 2021-10-06

**Authors:** Tina B. Marvasti, Faisal J. Alibhai, Lukasz Wlodarek, Anne Fu, Shu‐Hong Li, Jun Wu, Richard D. Weisel, Robert J. Cusimano, Maral Ouzounian, Terrence Yau, Ren‐Ke Li

**Affiliations:** ^1^ Division of Cardiovascular Surgery Peter Munk Cardiac Centre Toronto General Hospital Research Institute University Health Network Toronto Ontario Canada; ^2^ Division of Cardiovascular Surgery Department of Surgery University of Toronto Toronto Ontario Canada

**Keywords:** aging, bone marrow transplant, humanized mice, myocardial infarction

## Abstract

Ventricular remodeling following myocardial infarction (MI) is a major cause of heart failure, a condition prevalent in older individuals. Following MI, immune cells are mobilized to the myocardium from peripheral lymphoid organs and play an active role in orchestrating repair. While the effect of aging on mouse bone marrow (BM) has been studied, less is known about how aging affects human BM cells and their ability to regulate repair processes. In this study, we investigate the effect aging has on human BM cell responses post‐MI using a humanized chimeric mouse model. BM samples were collected from middle aged (mean age 56.4 ± 0.97) and old (mean age 72.7 ± 0.59) patients undergoing cardiac surgery, CD34^+/−^ cells were isolated, and NOD‐scid‐IL2rγ^null^ (NSG) mice were reconstituted. Three months following reconstitution, the animals were examined at baseline or subjected to coronary artery ligation (MI). Younger patient cells exhibited greater repopulation capacity in the BM, blood, and spleen as well as greater lymphoid cell production. Following MI, CD34^+^ cell age impacted donor and host cellular responses. Mice reconstituted with younger CD34^+^ cells exhibited greater human CD45^+^ recruitment to the heart compared to mice reconstituted with old cells. Increased cellular responses were primarily driven by T‐cell recruitment, and these changes corresponded with greater human IFNy levels and reduced mouse IL‐1β in the heart. Age‐dependent changes in BM function led to significantly lower survival, increased infarct expansion, impaired host cell responses, and reduced function by 4w post‐MI. In contrast, younger CD34^+^ cells helped to limit remodeling and preserve function post‐MI.

## Graphical abstract

To study the effect aging has on human cell responses post‐MI, we utilized a xenograft mouse (NSG) model using BM from middle aged (Y‐CD34+) and old patients (O‐CD34+). Our results demonstrate that O‐CD34+ reconstituted mice exhibit impaired donor and host cell responses while Y‐CD34+ reconstituted mice exhibit a greater donor lymphocyte and host myeloid cell responses post‐MI. Differences in cellular responses are associated with improved cardiac function in Y‐CD34+ reconstituted mice post‐MI.

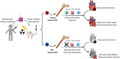

**Abbreviations**
BM: bone marrow
BMI: body mass index
CFU: colony‐forming unit
DAPI: 4′,6‐diamidino‐2‐phenylindole
hCD3^+^
: human T‐cell
hCD45: human CD45 cells
HSC: hematopoietic stem cells
IFN‐γ: human interferon‐γ
LAD: left anterior descending
M.O.M: Mouse on Mouse
MACS: magnetic activated cell sorting
MI: myocardial infarction
MMP9: metalloproteinase 9
NBF: neutral buffered formalin
NSG: NOD‐scid‐IL2rγ^null^
OCT: optical cutting temperature
PBS: phosphate‐buffered saline
PFA: paraformaldehyde
WGA: wheat germ agglutinin

## 1 INTRODUCTION

Successful infarct healing and scar formation is essential for preserving cardiac function following myocardial infarction (MI) (Prabhu & Frangogiannis, 2016). Although prompt reperfusion significantly reduces early mortality following acute MI, post‐ischemia heart failure is a major contributor to reduced quality of life, increased healthcare burden, and mortality worldwide (Cooper et al., 2015). One proposed approach for limiting adverse remodeling post‐MI has been stem cell transplantation. However, despite promising pre‐clinical findings, clinical studies of direct stem cell transplantation to the ischemic myocardium have yielded mixed results (Marvasti et al., 2019). Recent studies have demonstrated that effective cardiac cell therapy relies on modulation of endogenous repair processes, as the transplanted cells are only transiently present in the infarcted myocardium (Vagnozzi et al., 2020). Therefore, if endogenous repair mechanisms are impaired, such as in elderly patients or in patients with co‐morbidities (e.g., diabetes), it is anticipated that cell therapy will be less effective and less likely to yield beneficial results in these patient populations.

To overcome this limitation, our group has been investigating the potential of rejuvenation strategies to improve endogenous repair responses and stimulate cardiac repair post‐MI. We previously demonstrated that reconstituting aged mice with younger bone marrow (BM) hematopoietic stem cells (HSC) leads to stable integration of these cells and improves cardiac repair post‐MI (Li et al., 2017; Li et al., 2019; Tobin et al., 2020). This model is distinct from previous cell therapy approaches, as this approach requires stable engraftment of donor cells in the bone marrow which subsequently participate in the infarct healing process. Immune cells mobilize to the heart following infarction and orchestrate cardiac repair through several mechanisms. Although much is known about BM aging (Broxmeyer et al., 2020; Ho et al., 2019; Pang et al., 2011; Pritz et al., 2014), less is known about whether age‐dependent changes in human BM cells contribute to impaired cardiac repair post‐MI. Here, we establish a xenograft model to study the impact aging has on the function of human BM cells. We investigate the capacity of young and old patient BM cells to repopulate the BM of immune deficient NOD‐scid‐IL2rγ^null^ (NSG) mice and examine the ability of these cells to participate in infarct healing post‐MI.

## 2 RESULTS

### 2.1 Examination of human CD34^+^ cells frequency and function

**FIGURE 1:**
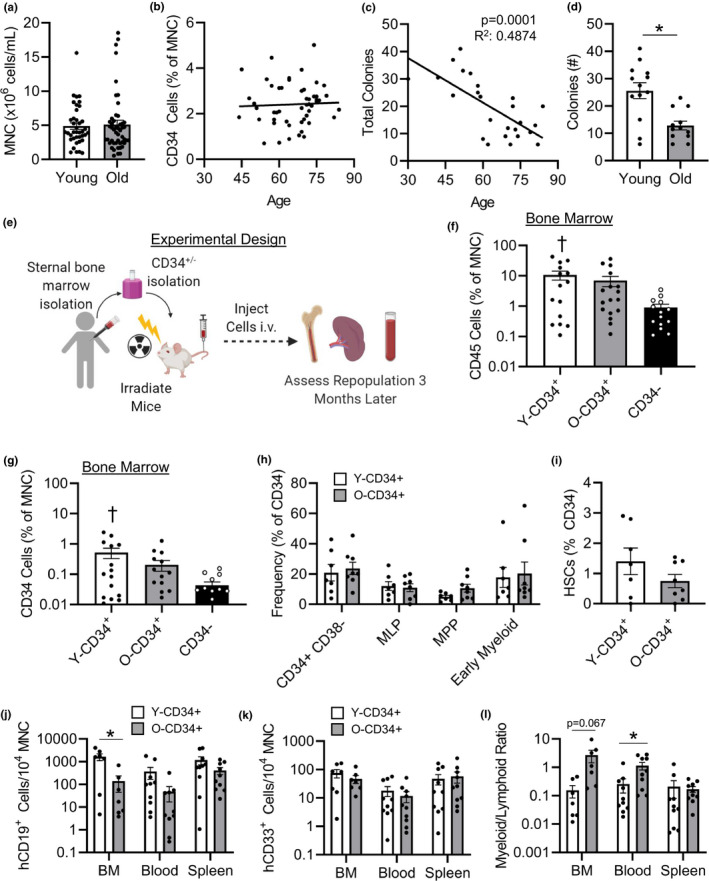
Old CD34^+^ cells exhibit reduced colony formation and lower lymphoid cell repopulation following transplant. (a) Mononuclear cell concentration (*n* = 40, 56.4 ± 0.97 years old [Y‐CD34+], and *n* = 48, 72.7 ± 0.59 years old [O‐CD34+]) and (b) CD34^+^ cell frequency in younger and old patient BM (*n* = 51, 56.8 ± 1.2 years old [Y‐CD34+] vs. 73 ± 0.7 years old [O‐CD34+]). (c) Colony‐forming ability of CD34^+^ cells from younger and old patient BM (*n* = 25, 52.7 ± 2.5 years old [Y‐CD34+] vs. 75.1 ± 1.4 years old [O‐CD34+]). (d) And total number of colonies formed stratified based on age group (*n* = 13, 52.7 ± 2.5 years old [Y‐CD34+] and *n* = 12, 75.1 ± 1.4 years old [O‐CD34+]). (e) Schematic representation of experimental design for reconstitution model. (f) hCD45^+^, and (g) CD34^+^ frequency in the BM at 3 months postreconstitution (*n* = 15, 56.2 ± 1.6 years old [Y‐CD34+], *n* = 17, 71.8 ± 0.7 years old [O‐CD34+], *n* = 14, 68 ± 2.6 years old [CD34^−^]). (h) Engrafted human hematopoietic progenitor cell frequencies in the BM of NSG mice transplanted with younger and old CD34^+^ bone marrow cells and (i) engrafted BM human HSCs 3 months after reconstitution (*n* = 7, 56.9 ± 1.4 years old [Y‐CD34+], *n* = 8, 73.1 ± 1.5 years old [O‐CD34+]). (j) Mice transplanted with younger CD34^+^ cells exhibited greater levels of B cells (CD19^+^) in the bone marrow 3 months post‐transplant; (k) myeloid cell frequency (CD33^+^) was similar between mice engrafted with younger and old cells and (l) myeloid (CD33^+^):lymphoid (CD19^+^) in the BM, blood, and spleen 3 months post‐reconstitution (*n* = 7–10/group, 57.6 ± 1.5 years old [Y‐CD34+] vs. 73.1 ± 1years old [O‐CD34+]).*p < 0.05 vs. indicated groups, ^†^ *p* < 0.05 vs. CD34^−^. Values are mean ± SEM

A total of 88 patients were included in this study and were separated into middle aged (mean age 56.4 ± 0.97) and old (mean age 72.7 ± 0.59) donors. Patients' characteristics were reviewed to evaluate any underlying differences between the two cohorts (Table 1). Analysis of complete blood counts prior to surgery demonstrated that old patients exhibited greater levels of circulating immune cells, mainly driven by increased neutrophils and monocytes (Figure S1a). However, total BM cellularity was not different between the middle aged and old cohorts (Figure 1a and Figure S1b). The frequency of CD34^+^ cells (Figure 1b and Figure S1c‐d), a common progenitor cell marker, and the frequency of BM lineage progenitor populations (Figure S1e‐h) were also similar in the middle aged and old patient cohorts. However, BM from older patients did exhibit an increased frequency of CD33^+^/CD14^+^ cells consistent with a greater frequency of myeloid cells in older individuals (Figure S2b). Although progenitor cell frequencies were similar, CD34^+^ progenitor cells exhibited an age‐dependent decline in colony formation capacity (Figure 1c) with significantly lower colonies formed with old versus middle aged CD34^+^ patient BM cells (Figure 1d). These data demonstrate that although the frequency of BM progenitor cells was similar between our middle aged and old patients, old cells exhibit impaired function.

**TABLE 1:** Patient characteristics and demographics

Patient demographics	Middle aged patients (*n* = 40)	Old patients (*n* = 48)	*p*‐value
Age	56.4 ± 0.97	72.7 ± 0.59	<0.0001
Male	33 (82%)	37 (77%)	0.60
Coronary artery disease	23 (57%)	33 (69%)	0.37
Valvular heart disease	19 (47%)	18 (37%)	0.39
Aortic disease	1 (2%)	1 (2%)	1.00
Smoking	10 (25%)	3 (6%)	0.02
Obese	15 (37%)	14 (29%)	0.49
Patient history			
Hypertension	29 (72%)	39 (81%)	0.44
Dyslipidemia	23 (57%)	29 (60%)	0.83
Angina	17 (42%)	32 (67%)	0.03
Diabetes mellitus	19 (47%)	23 (48%)	0.99
Myocardial infarction	9 (22%)	9 (19%)	0.79
Peripheral vascular disease	6 (15%)	8 (17%)	0.99
Medication history			
Anti‐hypertensive	29 (72%)	43 (89%)	0.06
Cholesterol lowering	27 (67%)	38 (79%)	0.23
Glucose lowering	16 (40%)	19 (39%)	0.99
Anti‐platelets	3 (7%)	7 (15%)	0.34
Anti‐coagulants	3 (7%)	7 (15%)	0.34
Nitroglycerin	4 (10%)	15 (31%)	0.02
Acetylsalicylic acid	26 (65%)	34 (71%)	0.65

Note

The categorical variables were assessed using Fisher's exact test and are presented as the absolute value and proportion in parentheses, and the continuous variables were assessed using Students' *t* test and are presented as mean ± SEM.

### 2.2 Aging reduces long‐term reconstitution potential and lymphoid cell production of human CD34^+^ cells

To assess the reconstitution potential of CD34^+^ cells in vivo, NSG mice were reconstituted with 7 x 10^5^ human CD34^+^ cells from middle aged or old patient donors, creating Y‐CD34^+^ and O‐CD34^+^ NSG chimera respectively (Figure 1e). CD34^−^ chimeras were created by reconstituting NSG mice with an equal concentration of cells from the CD34 depleted cell fractions (Figure S3a). Three months after reconstitution, examination of BM repopulation by flow cytometry revealed that younger CD34^+^ cells have the greatest repopulation potential, as indicated by the highest frequency in BM human CD45^+^ cells and human CD34^+^ cells (Figure 1f,g) in comparison with CD34^−^ cell group. A similar trend was also observed in the blood and spleen (Figure S3b). However, lineage progenitor frequencies were similar between younger and older CD34^+^ transplanted mice (Figure 1h). Hematopoietic stem cells trended to be more abundant in younger CD34+ transplanted mice (Figure 1i); however, this did not reach statistical significance as HSC engraftment was variable.

Further examination of the lineages produced by engrafted cells demonstrated that the lymphoid production capacity of younger CD34^+^ cells was significantly greater compared with old CD34^+^ cells, as mice transplanted with younger CD34^+^ cells exhibited greater levels of B cells (CD19^+^) in the BM 3 months post‐transplant (Figure 1j). B‐cell levels also trended higher in both the blood and spleen of NSG mice reconstituted with younger versus old CD34^+^ cells at baseline. Myeloid cell repopulation (CD33^+^) was similar between groups (Figure 1k); however, due to a reduction in lymphoid cell production, the myeloid (CD33^+^):lymphoid (CD19^+^) cell ratio was higher in NSG mice transplanted with old CD34^+^ versus younger CD34^+^ cells (Figure 1l). Progenitor cell engraftment and lineage production were not assessed in CD34^−^ due to the low number of cells engrafted at 3 months post‐transplant. Together, these data demonstrate that CD34^+^ cells from the middle aged patient cohort have increased colony formation in vitro, increased reconstitution potential in vivo, and greater lymphoid cell production in vivo compared with CD34^+^ cells from the old cohort.

### 2.3 Reconstitution of NSG mice with human CD34^+^ cells improves cardiac function post‐MI

**FIGURE 2:**
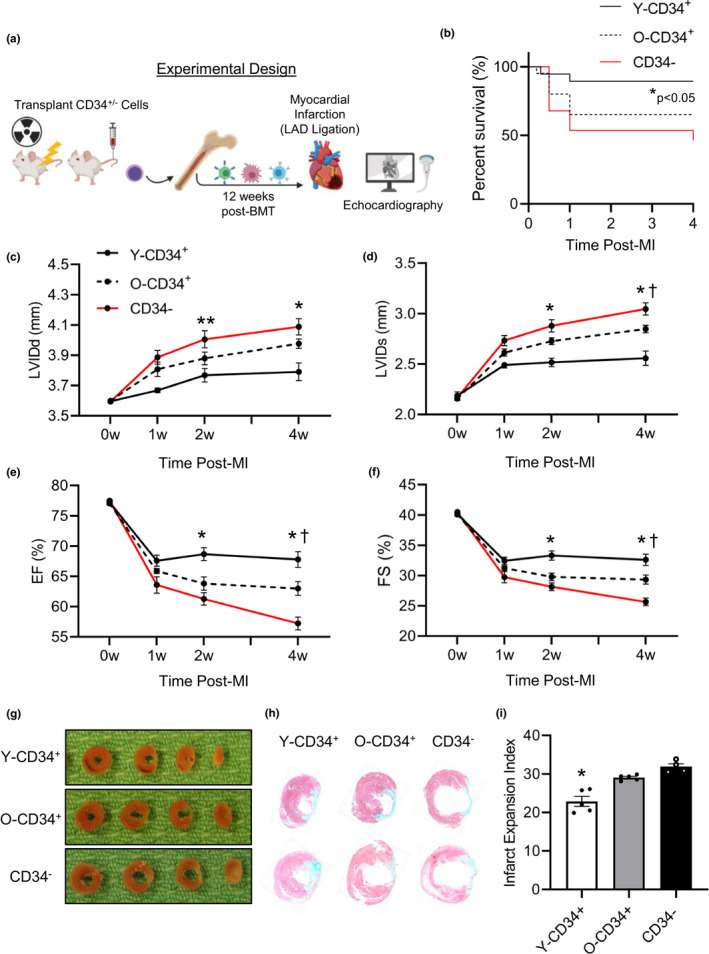
Age and cell type affect the long‐term functional outcome post‐MI. (a) Schematic representation of cardiac function analysis after reconstitution and coronary ligation. (b) Survival curve of Y/O‐CD34^+^ mice versus CD34^−^ mice by 4 weeks post‐MI, *p < 0.05 Y‐CD34^+^ vs. all groups. (c) Functional analysis post‐MI measured by echocardiography demonstrating dimensions at (c) diastole and (d) systole and cardiac function demonstrated by (e) ejection fraction and (f) fractional shortening at 4 weeks post‐MI (55.3 ± 2.1 years old [Y‐CD34+] vs. 74.1 ± 1.7 years old [O‐CD34+], *n* = 7‐10/group). All timepoint values are provided in Table S1. (g) Gross tissue and (h) histological analyses with trichrome staining at 4 weeks post‐MI for analysis of scar size. Two different stained hearts are shown for each group. (i) Quantification of infarct expansion comparison (53.6 ± 2.5 years old [Y‐CD34+] vs.73 ± 0.6 years old [O‐CD34+], *n* = 4‐5/group). *p < 0.05 vs. Y‐CD34^+^ all groups at same time point, ^†^ *p* < 0.05 O‐CD34^+^ vs. CD34^−^ at same time point, and **p < 0.05 Y‐CD34^+^ vs. CD34^−^ at same time point. Values are mean ± SEM

Next, we assessed whether aging impairs the ability of human BM cells to participate in cardiac repair post‐MI using an in vivo coronary artery ligation model. Three months after reconstitution, NSG mice were infarcted (Figure 2a). Examination of early survival post‐MI revealed that Y‐CD34^+^ mice exhibit significantly greater survival in the first week post‐MI compared to O‐CD34^+^ and CD34^−^ mice (Figure 2b). Post‐mortem investigation of mice indicated ventricular rupture as the main cause of death post‐MI.

Next, we examined cardiac function using echocardiography over a 4‐week period. (Table S1). Y‐CD34^+^ mice exhibited significantly reduced ventricular remodeling post‐MI indicated by significantly reduced dimensions at diastole and systole (Figure 2c,d). Consistent with reduced remodeling, Y‐CD34^+^ mice had significantly greater cardiac function indicated by higher ejection fraction and fractional shortening at 4 weeks post‐MI (Figure 2e,f). Analysis of the akinetic wall region in the LV revealed animals reconstituted with Y‐CD34^+^ cells exhibit the smallest size of dysfunctional tissue, while O‐CD34^+^ cells also offered some beneficial effects compared with CD34^−^ hearts (Figure S3c). Further examination of the cardiac tissue by morphological (Figure 2g) and histological analyses (Figure 2h,i) at 4 weeks post‐MI confirmed reduced left ventricular remodeling in Y‐CD34^+^ hearts, as trichrome staining demonstrated that Y‐CD34^+^ hearts had significantly less infarct expansion compared to their O‐CD34^+^ and CD34^−^ counterparts (Figure 2h,i). Although Y‐CD34^+^ mice exhibit the greatest improvement in outcome, old CD34^+^ cells appear to offer some benefits as cardiac function was significantly greater compared to CD34^−^ mice at 4 weeks post‐MI. To further understand the effect reconstituted CD34^+^ cells have on the repair process, we also compared the functional outcome of reconstituted mice to WT NSG mice which were irradiated at 285cGy 3 months prior to LAD ligation but did not receive BM transplant (Figure S4). LV dilation and cardiac function of WT NSG mice was not different from O‐CD34^+^ and CD34^−^ mice at 4 weeks post‐MI and was significantly worse than Y‐CD34+ mice. This is consistent with a beneficial effect of Y‐CD34^+^ cells. Collectively, these data demonstrate that NSG mice engrafted with younger CD34^+^ cells exhibit significantly better functional outcome and survival post‐MI.

### 2.4 Y‐CD34^+^ cells enhance cardiac repair and minimize remodeling post‐MI

**FIGURE 3:**
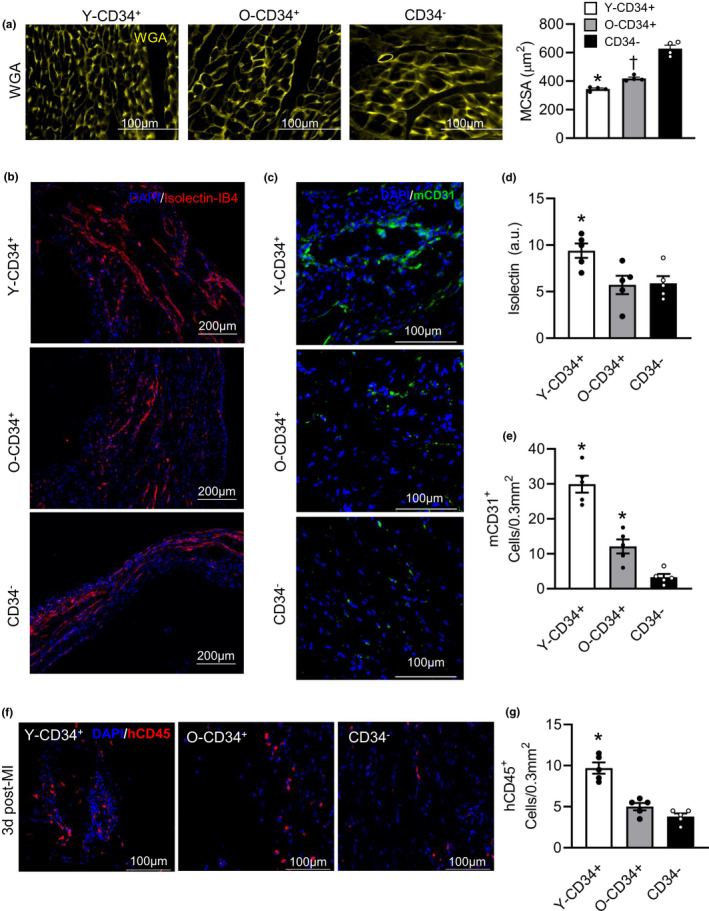
Younger CD34^+^ cells reduce remodeling and improve scar angiogenesis by 4 weeks post‐MI. (a) WGA staining of younger CD34^+^, old CD34^+,^ and CD34^−^ myocytes for cross‐sectional area analysis at 4w post‐MI, and quantification (52.2 ± 3.0 years old [YCD34+] vs. 71.2 ± 1.6 years old [O‐CD34+], vs. 59.2 ± 2.9 years old [CD34‐], *n* = 4/group). (b) Isolectin‐IB4 staining of the infarct region. (c) Mouse CD31^+^ cell staining in the infarct regions. (d) Quantification of isolectin staining for comparison between the three groups (50.6 ± 2.8 years old [Y‐CD34+] vs. 70.8 ± 1.3 years old [O‐CD34+], vs. 57.4 ± 2.1 years old [CD34‐], *n* = 5/group). (e) Quantification of mCD31 cell staining for angiogenesis comparison between the groups (50.6 ± 2.8 years old [Y‐CD34+] vs. 70.8 ± 1.3 years old [O‐CD34+], *n* = 5/group). (f) Human CD45^+^ staining demonstrating inflammatory cell infiltration into the myocardial infarct region at 3d post‐MI. (g) Quantification of the hCD45^+^ staining at 3d post‐MI among the younger CD34^+^ (50.6 ± 2.8 years old), old CD34^+^ (70.8 ± 1.3 years old), and CD34^−^ hearts (*n* = 5/group). *p < 0.05 vs. all groups, ^†^ *p* < 0.05 vs. CD34^−^. Values are mean ± SEM

To assess how CD34^+^ cells benefit infarct healing, we evaluated tissue remodeling and angiogenesis at 4 weeks post‐MI. First, examination of cardiomyocyte cross‐sectional area (CSA) demonstrated that following infarction remote zone cardiomyocyte CSA is greatest in CD34^−^ mouse hearts consistent with increased remodeling (Figure 3a). Cardiomyocyte CSA in both Y‐CD34^+^ and O‐CD34^+^ hearts was smaller than CD34^−^ (Figure 3a), and myocytes were smallest in Y‐CD34^+^ hearts. Analysis of angiogenesis in the damaged myocardium through quantification of isolectin‐IB4, a marker of capillary density, demonstrated that Y‐CD34^+^ hearts have greater staining within the infarcted myocardium compared to old CD34^+^ and CD34^−^ cells, suggesting improved scar vascularization in this group (Figure 3b,d). Further examination of mouse CD31^+^ cells revealed greater cell density in the peri‐infarct region of Y‐CD34^+^ hearts compared to O‐CD34^+^ hearts, supporting improved scar vascularization in these mice (Figure 3c,e). Human CD31 cells were a rare cell population within the infarcted myocardium and were present in few samples, suggesting that these cells do not contribute to the vascularization process (Figure S3d). Overall, these data indicate that engrafted human CD34^+^ cells modulate the infarct healing process post‐MI to improve cardiac repair post‐MI. Moreover, they demonstrate that Y‐CD34^+^ cells more effectively reduce remodeling and stimulate infarct healing compared to old CD34^+^ cells.

### 2.5 Human immune cell recruitment post‐MI is reduced with age

**FIGURE 4:**
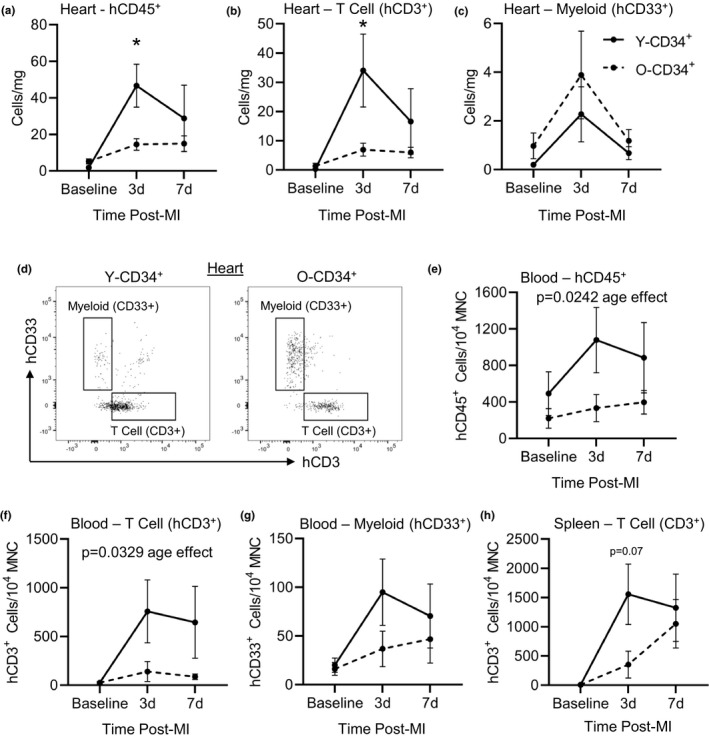
Aged CD34^+^ cells exhibit impaired human cell recruitment to the heart post‐MI. (a) Human CD45 cells (hCD45), (b) human T cells (hCD3), and (c) human myeloid (hCD33) cells in the mouse myocardium at baseline, 3, and 7 d post‐MI as measured by flow cytometry (56.5 ± 3.8 years old [Y‐CD34+] vs. 72.2 ± 0.8 years old [O‐CD34+] at baseline, 57 ± 1.2 years old [Y‐CD34+] vs. 71.9 ± 1.0 years old [O‐CD34+] at 3 d and 55.4 ± 1.7 years old [Y‐CD34+] vs. 72.7 ± 1.2 years old [O‐CD34+] at 7 d post‐MI, *n* = 4–10/group). (d) Representative flow cytometry plots showing differences in T‐cell cell number. (e) Circulatory hCD45^+^, (f) hCD3^+^, (g) CD33^+^ cell frequency in the blood of Y/O‐CD34^+^ reconstituted mice at baseline, 3 and 7 d post‐MI (56.5 ± 1.6 years old [Y‐CD34+] vs. 73.1 ± 1.0 years old [O‐CD34+] at baseline, 56.8 ± 0.7 years old [Y‐CD34+] vs. 72.2 ± 1.3 years old [O‐CD34+] at 3 d, and 58 ± 1.4 years old [Y‐CD34+] vs. 72.4 ± 1.1 years old [O‐CD34+] at 7 d post‐MI, *n* = 7–10/group). (h) Spleen hCD3^+^ cell frequency in chimeric mice at baseline, 3 and 7 d post‐MI (56.5 ± 1.6 years old [Y‐CD34+] vs. 73.1 ± 1.0 years old [OCD34+] at baseline, 56.5 ± 0.7 years old [Y‐CD34+] vs. 72.2 ± 1.3 years old [O‐CD34+] at 3 d, and 58.4 ± 1.4 years old [Y‐CD34+] vs. 72 ± 1.1 years old [O‐CD34+] at 7 d post‐MI, *n* = 7–10/group). *p < 0.05 between groups at indicated time point. Values are mean ± SEM

Next, we examined early cellular responses post‐MI. First, we assessed human CD45^+^ cell recruitment at 3 days (3d) post‐MI by immunofluorescence to determine whether human immune cells also infiltrate the infarct post‐MI. Interestingly, we found human cells in all groups, and Y‐CD34^+^ hearts had the highest levels of human cells. Human CD45^+^ cells were significantly higher in the infarcted myocardium in Y‐CD34^+^ hearts compared with O‐CD34^+^ and CD34^−^ hearts at this time point (Figure 3f,g). These cells were confirmed to be of human origin using a second marker, Ku80 (Figure S3d). In order to further understand the sub‐populations of recruited immune cells, we examined human cell responses post‐MI by flow cytometry at 3d and 7d post‐MI. Examination of human CD45 cells (hCD45) in the heart revealed that O‐CD34^+^ mice exhibit less CD45^+^ cells in the infarcted heart post‐MI compared to Y‐CD34^+^ mice (Figure 4a), consistent with our staining analysis at this time point (Figure 3f,g). Analysis of hCD45^+^ sub‐populations revealed that a greater human T‐cell response (hCD3^+^) in Y‐CD34^+^ mice at 3d post‐MI was primarily responsible for increased hCD45^+^ cell recruitment (Figure 4b,d). By 7d post‐MI, CD3^+^ numbers declined in Y‐CD34^+^ hearts indicating resolution of the inflammatory phase. Recruitment of myeloid (CD33^+^) and B‐cell (CD19^+^) populations were not different between Y‐CD34^+^ and O‐CD34+ mice post‐MI (Figure 4c and Figure S5a).

To understand why Y‐CD34^+^ and O‐CD34^+^ mice exhibit different responses, we examined human immune cell populations in the blood and spleen post‐MI. A blunted immune response in O‐CD34^+^ mice corresponded with reduced mobilization of immune cells into the circulation post‐MI compared with Y‐CD34^+^ (Figure 4e). This was primarily driven by differences in T‐cell mobilization in into the circulation. Although human T‐cell levels were low in baseline reconstituted animals, infarction led to an increase in circulating cell numbers, and this increase was greatest in Y‐CD34^+^ mice (Figure 4f). A similar trend was observed with myeloid cells, but this was not significantly different between Y‐CD34^+^ and O‐CD34^+^ mice (Figure 4g). Interestingly, changes in the circulation mirrored the temporal cell responses in the spleen. Post‐MI Y‐CD34^+^ mice exhibited a greater expansion of T cells, as spleen human CD3+ cell number was significantly higher compared to baseline spleens and trended greater compared to O‐CD34^+^ spleens at 3d post‐MI (Figure 4h). In contrast O‐CD34^+^ mice exhibit a delayed T cell response, as expansion of spleen CD3^+^ cells was observed by 7d post‐MI. B‐cell frequency was largely unaffected and was not significantly different between reconstituted mice post‐MI (Figure S5b,d).

### 2.6 Human CD34^+^ cells alter cytokine and MMP9 responses post‐MI

**FIGURE 5:**
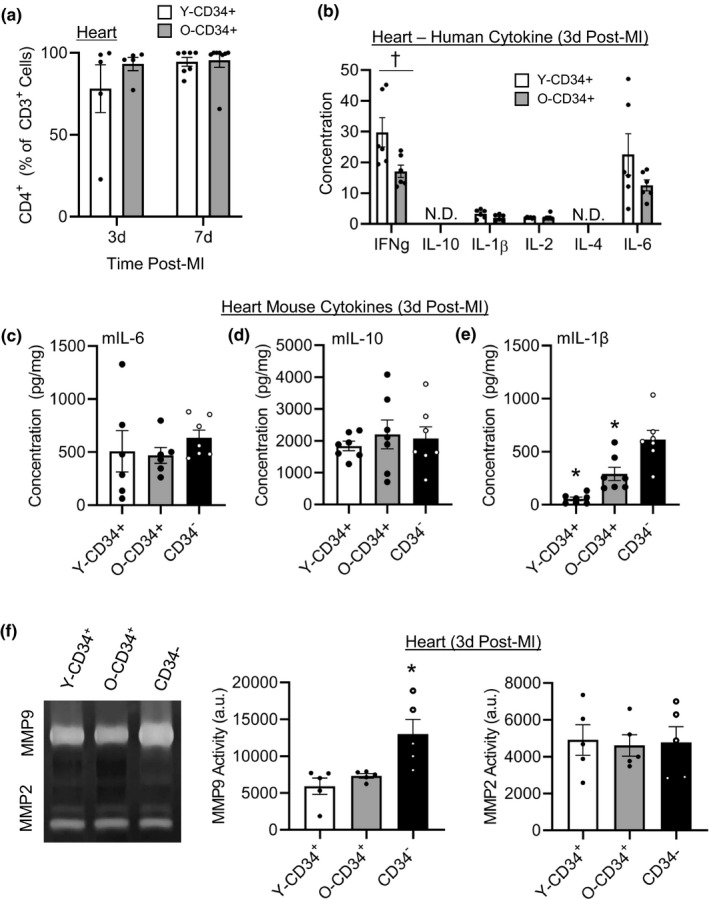
CD34^+^ cell age affects early mediators of cardiac repair. (a) T‐cell subtype analysis of CD4^+^ frequency 3 and 7 d post‐MI (58.2 ± 1.9 years old [Y‐CD34+] vs. 71.9 ± 1.0 years old [O‐CD34+] at 3 d and 57 ± 1.6 years old [Y‐CD34+] vs. 73 ± 0.9 years old [OCD34+] at 7 d post‐MI, *n* = 5–8/group). (b) Human cytokine analysis of pro‐ and anti‐inflammatory cytokines in younger and older CD34^+^ hearts at 3 d post‐MI (56 ± 2.1 years old [Y‐CD34+] vs. 69.5 ± 1.7 years old [O‐CD34+], *n* = 6/group). Myocardial mouse cytokines (c) IL‐6, (d) IL‐10, and (e) IL‐1β in Y/O CD34^+^ and CD34^−^ myocardium (56 ± 2.1 years old [Y‐CD34+] vs. 69.5 ± 1.7 years old [O‐CD34+], *n* = 7/group). (f) MMP2 and MMP9 activity at 3 d post‐MI in in Y/O CD34^+^ and CD34^−^ infarcted myocardium measured using zymography (55.6 ± 2.2 years old [Y‐CD34+] vs. 69.8 ± 1.9 years old [O‐CD34+], *n* = 5/group). *p < 0.05 vs all other groups, ^†^ *p* < 0.05 groups as indicated^−^. Values are mean ± SEM

Subtype analysis indicated that the T cells which infiltrate the heart post‐MI are primarily T‐helper cells, CD4^+^ (Figure 5a and Figure S5e). Thus, we next assessed human cytokine levels in the heart at 3d post‐MI to determine whether cytokine abundance corresponded with changes cell infiltration. Consistent with greater T‐cell levels in Y‐CD34^+^ hearts, human interferon‐γ (IFN‐γ) was significantly elevated compared with O‐CD34^+^ hearts at 3d post‐MI (Figure 5b). Human IL‐10 and IL‐4 were below the limits of detection, and human IL‐1β and IL‐2 were detected in low quantities and not different between Y‐CD34^+^ and O‐CD34^+^ hearts. Human IL‐6 was higher in Y‐CD34^+^ hearts; however, the response was variable as not statistically different between groups. We also assessed whole heart mouse cytokine levels at 3d post‐MI to understand how human CD34^+^ cells affect host infarct healing responses. While TNFα was below the limit of detection, IL‐6 (Figure 5c), IL‐10 (Figure 5d), and IL‐1β were detectable (Figure 5e). IL‐6 and IL‐10 were similar between all groups however, IL‐1β was significantly affected by cell type and CD34^+^ cell age. O‐CD34^+^ hearts had significantly lower IL‐1β levels compared with CD34^−^ hearts, while Y‐CD34^+^ hearts had the lowest expression of IL‐1β and was significantly lower compared with O‐CD34^+^ and CD34^−^ hearts (Figure 5e). We also assessed matrix metalloproteinase 9 (MMP9) in the infarcted myocardium as this is a key factor which mediates remodeling post‐MI. Gelatin zymography revealed increased MMP9 activity level in CD34^−^ hearts compared with Y‐CD34^+^ and O‐CD34^+^ hearts (Figure 5f). This is consistent with increased remodeling in this group. MMP2 levels were not different between groups. Collectively, these data demonstrate that Y‐CD34^+^ mice exhibit a greater human cell response primarily driven by the mobilization of T cells.

### 2.7 Impact of transplanted cells on host cell responses post‐MI

**FIGURE 6:**
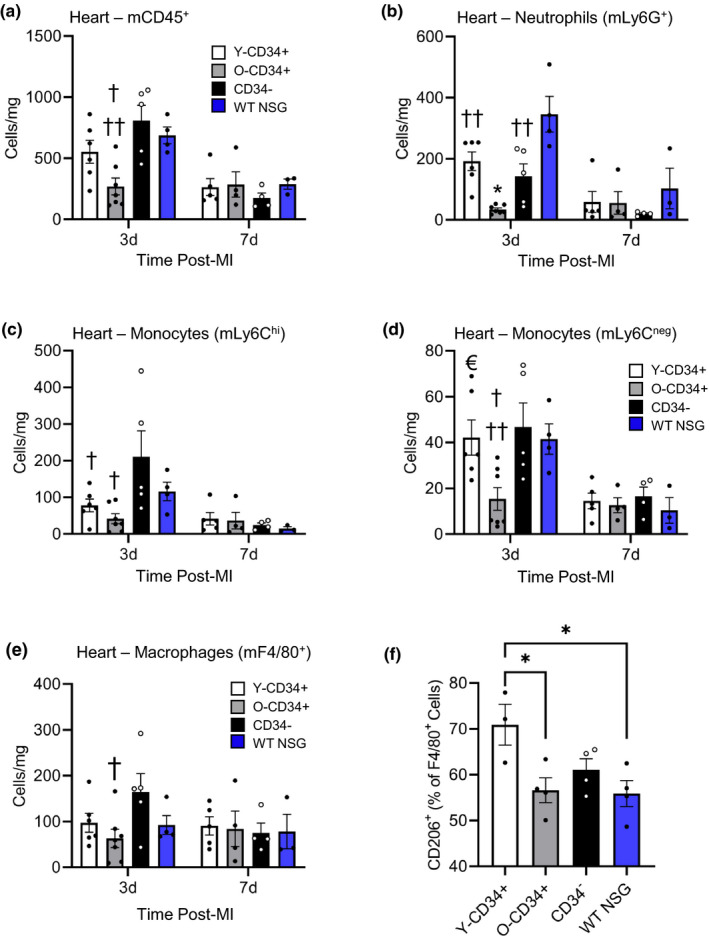
CD34^+^ cell age alters the infiltration of mouse (host) myeloid cells post‐MI. (a) Quantification of mouse CD45^+^ (mCD45) cells in the heart 3‐ and 7 d post‐MI (56.3 ± 1.1 years old [Y‐CD34+] vs. 70.1 ± 1.6 years old [O‐CD34+], vs. 68.5 ± 3.5 years old [CD34‐] at 3 d and 61.5 ± 1.4 years old [Y‐CD34+] vs. 70.3 ± 1.4 years old [O‐CD34+], vs. 65.5 ± 3.1 years old [CD34‐] at 7 d, *n* = 3–7/group). Quantification of mouse (b) neutrophil, (c) Ly6C^hi^ monocytes, and (d) Ly6C^neg^ monocytes 3 and 7 d post‐MI in Y‐CD34^+^, O‐CD34^+^, CD34^−^, and WT NSG mice (*n* = 3–7/group). (e) Quantification of total macrophages in heart 3‐ and 7‐d post‐MI and (f) frequency of CD206^+^ macrophages in the heart at 3 d post‐MI. ^†^ *p* < 0.05 vs. CD34^−^ at the same timepoint, ^††^ *p* < 0.05 vs. WT NSG at the same timepoint, *p < 0.05 vs indicated groups at same timepoint, €*p* < 0.05 vs. O‐CD34^+^ at the same timepoint. Values are mean ± SEM

Although human cells are differentially recruited, our flow analyses indicate that the transplanted cells are a relatively smaller population compared to the host (mouse) cell population within the bone marrow 3 months after reconstitution (Figure S2c). Therefore, we next examined whether the age of CD34^+^ cells can affect mouse cell recruitment post‐MI. To do this, we first examined mouse CD45^+^ cells in the infarcted myocardium 3d post‐MI using immunofluorescence (Figure S6). Responses were compared to animals that were irradiated at 285cGy 3 months prior to ligation but did not receive bone marrow transplant (no reconstitution; WT NSG) to determine the effect of bone marrow transplant on host cell responses. Quantification revealed that the number of host (mouse) CD45^+^ (mCD45^+^) cells in the infarct region of Y‐CD34^+^ and O‐CD34^+^ reconstituted mice was significantly lower than in the hearts of CD34^‐^ reconstituted and WT NSG mice. In light of these findings, we next examined the recruitment of mouse immune cell sub‐populations to the heart 3d and 7d post‐MI using flow cytometry. Consistent with the temporal nature of the immune response post‐MI, mouse CD45^+^ cells were more abundant at Day 3 post‐MI versus Day 7 (Figure 6a).

Interestingly, the cellular response in O‐CD34^+^ mice was significantly lower compared to CD34^−^ and WT mice (Figure 6a). This was driven by a reduction in the number of infiltrating neutrophils (Figure 6b) and monocytes (Figure 6c,d). Neutrophil cell numbers were also lower in the hearts of Y‐CD34^+^ and CD34^−^ mice compared with WT NSG mice. Reconstitution with Y‐CD34^+^ cells was also associated with a significantly reduced number of pro‐inflammatory Ly6C^hi^ monocytes in the heart at 3d post‐MI compared to CD34^−^ hearts. Ly6C^hi^ cell numbers in Y‐CD34^+^ mice were similar to O‐CD34^+^ mice. In contrast, Ly6C^neg^ monocytes were only affected by O‐CD34^+^ reconstitution, as these mice exhibited fewer cells in the heart at 3d post‐MI (Figure 6d). Consistent with lower monocyte infiltration, O‐CD34^+^ hearts had fewer F4/80^+^ macrophages compared to CD34^−^ hearts at 3d post‐MI (Figure 6e). We further examined potential macrophage phenotypes using CD206 as a marker of M2 polarization. Y‐CD34^+^ hearts exhibited a significantly greater frequency of CD206^+^ macrophages at 3d post‐MI compared to OCD34^+^ and WT NSG hearts. Collectively, these data demonstrate that O‐CD34^+^ reconstituted mice exhibit reduced host cell responses post‐MI leading to less immune cell infiltration. In contrast, Y‐CD34^+^ cells limit pro‐inflammatory Ly6C^hi^ monocyte and neutrophil cell infiltration, while not affecting the Ly6C^neg^ monocyte population.

To further assess the impact human cells have on mouse cell responses post‐MI we examined Ki67^+^ cells in the spleen 3d post‐MI, as a marker of cell proliferation (Figure S8a). Consistent with our flow cytometry analysis, Y‐CD34^+^ mice had the highest number of human CD45^+^ cells in the spleen at this time point (Figure S8b). Y‐CD34^+^ spleens also had a significantly greater number of Ki67^+^/hCD45^+^ cells compared with O‐CD34^+^ spleens, suggesting a higher proliferation rate in these cells (Figure S8c). Host cells were also impacted by human CD34^+^ cells, as Y‐CD34^+^ spleens had a greater number of mouse Ki67^+^ cells compared to O‐CD34^+^ spleens (Figure S8d). Interestingly, WT NSG cells showed the greatest level of proliferation suggesting that reconstitution with human cells impacts mouse cell responses. This is unlikely to be related to the irradiation as the WT NSG mice were also irradiated at 285cGy 3 months prior to ligation. Instead, this data demonstrates that O‐CD34^+^ cells have the greatest impact on host cell proliferation, suggesting that these cells negatively influence host cell responses post‐MI.

## 3 DISCUSSION

Aging is associated with a decline in myocardial repair; thus, understanding cellular deficiencies in older patients is an important step for developing targeted therapies which can improve repair post‐MI. However, the majority of pre‐clinical studies have been performed using young rodent models and little is known about how aging affects human BM responses post‐MI. To study this, we utilized a human xenograft mouse model of BM reconstitution using BM from middle aged and old patients. Consistent with previously published work, old CD34^+^ cells exhibited reduced colony formation in vitro (Kuranda et al., 2011). Moreover, in our NSG BM transplant model, we demonstrated that the old CD34^+^ cells have reduced repopulation with lower lymphoid cell production, consistent with previously published studies (Pang et al., 2011). Ultimately, these differences contribute to greater mortality in aged cell chimeric mice during the early phase post‐MI and significantly impaired long‐term cardiac function in comparison with the mice reconstituted with Y‐CD34^+^ human cells. To understand the possible reason, we performed a post‐mortem analysis and found great ventricular rupture as the main cause of death post‐MI. The beneficial effect of Y‐CD34^+^ cells on cardiac repair was also evident in our long‐term cardiac function analysis as mice reconstituted with Y‐CD34^+^ cells demonstrated the greatest tissue repair with lower ventricular dilation and greater cardiac function than mice reconstituted with old CD34^+^ cells or CD34^−^ cells at 4 weeks post‐MI. Reduced remodeling in Y‐CD34^+^ mice correlated with greater scar angiogenesis, reduced infarct expansion, and lower remote zone hypertrophy at 4 weeks post‐MI. We conducted multiple cytokine and cellular analyses in the infarcted myocardium to identify differences in the early repair processes that may explain differences in functional outcome post‐MI. We also assessed how the age of cells used for reconstitution affected host responses post‐MI. Together, our cytokine, flow cytometry, and immunofluorescence data suggest that old CD34^+^ cells negatively impact mouse repair responses post‐MI, while Y‐CD34^+^ cells show less of an inhibitory effect on host responses in addition to exhibiting a greater human CD3^+^ lymphocyte response post‐MI. These differences led to a significantly different cardiac cellular composition between Y‐CD34^+^ and O‐CD34^+^ reconstituted animals post‐MI. These findings suggest that human cells cross talk with host mouse cells to influence their responses post‐MI and suggest that cells derived from O‐CD34^+^ cells negatively influence surrounding cells to limit their responses following injury, such as following myocardial infarction.

While investigating cell responses post‐MI we found that mice reconstituted with Y‐CD34^+^ cells exhibited greater human T‐cell recruitment to the infarcted myocardium by 3d post‐MI. This paralleled expansion in the spleen, mobilization to the blood, and increased human IFNγ in the infarcted myocardium at 3d post‐MI. In contrast, mice reconstituted with old CD34^+^ cells exhibited blunted T‐cell recruitment to the blood and infarcted myocardium, delayed expansion of T cells in the spleen, and reduced IFNγ in the infarcted myocardium. In recent years, T cells have been shown to be an essential component of the infarct healing response post‐MI. Moreover, clinical studies have demonstrated that lymphocytes may play a role in infract repair as activation was observed in patients post‐MI (Cheng et al., 2005; Moraru et al., 2006). Pre‐clinical studies have provided additional mechanistic insights into the role these cells have in cardiac repair post‐MI. For example, using CD4^+^ T cell deficient mice Hofmann and colleagues investigated the role CD4^+^ T cells play in the repair processes post‐MI (Hofmann et al., 2012). This study demonstrated that CD4^+^ T cells act to limit pro‐inflammatory cell and cytokine responses in the heart, which together limit cardiac remodeling in mice. More recent studies have shown that activation of CD4^+^ T cells post‐MI are due to the release of cardiac antigens which are subsequently presented to these cells and active them in an antigen‐dependent manner (Rieckmann et al., 2019). In the setting of MI, this response does not lead to a detrimental auto‐immune response (as in the case with myocarditis), but instead helps to modulate the immune response, limit remodeling, and stimulate scar formation (Rieckmann et al., 2019). Within the T‐cell population, specific sub‐populations have been shown to influence cardiac repair post‐MI. For example, CD4^+^ FOXP3^+^ T_reg_ cell delivery has been shown to reduce infarct size and attenuate MI‐induced cardiac remodeling post‐ischemia in mice (Sharir et al., 2014). In another study, ablation of T_reg_ cells, using a Foxp3^DTR^ model, prior to MI resulted in increased infarct size, left ventricular dilation, and worse functional outcome post‐MI (Weirather et al., 2014). Future studies investigating the effect aging has on the activation and recruitment of T cell subsets post‐MI will help elucidate the role these cells have in cardiac repair in aged individuals.

Although the molecular mechanisms underlying the beneficial effects of human CD4^+^ T cells require further investigation, one hypothesis is that CD4^+^ cells infiltrate the myocardium post‐MI and modulate the cytokine/chemokine milieu to influence neighboring cell function. In this study, we show that mice reconstituted with Y‐CD34^+^ cells exhibit greater T‐cell infiltration and IFNγ protein levels in the infarcted myocardium at 3d post‐MI. Although cytokine signaling pathways have been shown to regulate myocardial repair post‐MI, the role of IFN‐y in early repair process remains largely unknown. Recently, Finger et al. (2019) suggested that IFNγ plays a protective role post‐MI, as IFN‐y deficient mice showed reduced survival and worse cardiac function post‐MI. Using an IFNγ reporter, the authors also identified lymphocytes as a key source of IFN‐y in the heart during early inflammatory phase post‐MI. Interestingly, the loss of IFN‐y was associated with lower neutrophil and monocyte cell numbers in the infarcted myocardium 3d post‐MI, suggesting that this cytokine plays a role in the recruitment of these cells. Future time course studies assessing human and mouse cytokine expression in reconstituted NSG mice post‐MI will help reveal how differences in cardiac cell composition affect the temporal expression of these key mediators of repair. In the context of aging, T cells have been shown to interact with other cell types in the heart and influence their function. For example, adoptive transfer of aged T cells into NSG‐DR1 mice alters the cardiac transcriptome and increases the infiltration of monocytes and macrophages into the heart (Delgobo et al., 2021). These findings are consistent with general adverse effects of aged T cells on organism fitness, as premature aging of T cells results in reduced animal longevity as well as a decline in the function of multiple organ systems (Desdin‐Mico et al., 2020). Our study is consistent with these findings as we demonstrate that aged cells can alter the function of host cells (young NSG mouse cells). In addition, we also show that greater T‐cell responses in Y‐CD34^+^ mice are associated with reduced mouse IL‐1β levels in the heart and that these changes correlate with greater survival, improved infarct healing, and better cardiac function post‐MI.

Therefore, our findings are twofold as our results suggest that old CD34^+^ cells negatively impact mouse repair responses post‐MI while Y‐CD34^+^ cells show less of an inhibitory phenotype and have a greater lymphocyte response; this ultimately contributes to improved cardiac repair post‐MI compared to reconstituted and WT NSG mice. These findings are consistent with our group's previous studies showing that the reconstitution of old mice with young bone marrow cells improves tissues repair processes. For example, our laboratory has shown that young mouse bone morrow cells can affect numerous physiological processes, as transplantation into aged mice improves cardiac repair post‐MI (Li et al., 2019), alters circulating factors (Alibhai et al., 2020), improves skeletal muscle repair following injury (Tobin et al., 2021), and improves learning and memory (Wlodarek et al., 2020). Bone marrow‐derived cells are in constant communication with peripheral tissues and interact with multiple organ systems during their lifespan. Considering the systemic nature of these cells, these cells have the capacity to influence numerous organ systems and thus are an interesting target for rejuvenation therapies (Alibhai & Li, 2020; Marvasti et al., 2019). Our findings in this study demonstrate the impact aging has on human bone marrow cell function and also suggest that rejuvenation of lymphoid populations such as T cells may yield beneficial effects for tissue repair in older patients. It is important to note that the BM‐derived T cells in our study are a likely a different population of cells compared to those obtained from the peripheral circulation. T cells in our study arise solely from BM stem cells/progenitors, while circulating T‐cell levels are derived from a balance between thymic output and peripheral proliferation. Future studies investigating the effect that the adoptive transfer of T cells obtained from the peripheral circulation or secondary lymphoid organs has on cardiac repair post‐MI will help elucidate how aging affects the function of T cells from different compartments. Moreover, rejuvenation therapies which aim to improve T‐cell function may require targeting lymphoid organs such as the thymus and lymph nodes.

As with any investigation involving a patient population, there are inherent limitations to this study. Here, the patient cohort was divided into “Middle Aged” or “Old” groups. In this investigation, the average age of the “Middle Aged” cohort is ~56 years old and the average age of the “Old” cohort is ~73 (Table 1). It is possible that the differences observed may be even more drastic if the “Middle Aged” cohort were 20–30 years of age, as in other investigations studying the effect aging has on BM function (Kuranda et al., 2011; Pang et al., 2011). However, our results support the notion that aging impairs BM cell function which leads to the production of cells that are less capable of stimulating cardiac repair post‐MI. Future therapies which restore the function of aged BM cells may restore immune and angiogenic responses and reduce extensive remodeling following ischemic injury in older patients.

## 4 EXPERIMENTAL PROCEDURES

### 4.1 Patient selection criteria

The research ethics board of the University Health Network approved the investigation. This study also complies with the Declaration of Helsinki. Male and female patients older than 30 years of age who were scheduled for non‐emergency cardiac surgery including coronary artery bypass graft or valve surgery or both were consented for the study. Ninety patients were recruited at the Toronto General Hospital between 2016 and 2019. Out of the 90 recruited patients, 2 were excluded from the study due to congenital cardiac abnormalities (congenital pulmonary valve stenosis and tetralogy of Fallot). Informed written consent was given prior to the inclusion of subjects in the study. Patients with pre‐operative intra‐aortic balloon pump, left ventricular assist device, extracorporeal membrane oxygenator, or previous history of cardiac surgery were excluded from the study. Patients with known active malignancy within the past 3 years or simultaneous participation in another study with an investigational pharmacological agent were not recruited. There was no patient follow‐up. Patients were considered smokers if they were actively smoking at the time of recruitment or quit smoking less than a year prior. Patients were considered obese if their body mass index (BMI) was greater than or equal to 30. Patient medication history was collected from the patient charts and detailed in Table 1. Cholesterol lowering medications include statins and ezetimibe. Glucose lowering medications include metformin and insulin.

### 4.2 Sternal bone marrow harvest

On the day of the surgery, an 18‐gauge needle containing 5 ml of 10% heparin solution was advanced slowly through the periosteum of the sternum and rotated as it passed through the anterior table. The solution was injected into the sternum, and 5–20 ml of bone marrow fluid was aspirated from the sternum. Fresh human bone marrow cells aspirated from the sternum during thoracotomy were transferred to the laboratory for cell isolation. Fresh human mononuclear cells were isolated using the Ficoll gradient (17144003, GE Healthcare), rinsed, and prepared for CD34+ cell sorting using the magnetic activated cell sorting (MACS) using a commercially available kit (Stem Cell Technologies, 17856). Immediately following isolation cells were counted and 0.7 x 10^6^ human CD34^+^ or CD34^−^ sorted cells were injected to the vein tail of irradiated NSG mice.

### 4.3 CD34 cell isolation and hematopoietic colony‐forming unit assay

Fresh mononuclear cells were separated into positively and negatively labeled CD34 fractions using magnetic activated cell sorting (MACS; Cat# 18056, Stem Cell Technologies). The purity of cells was assessed by flow cytometry. Functional capacity of CD34^+^ stem cells derived from the patients was evaluated using the colony‐forming unit (CFU) assay for human hematopoietic cells (MethoCult H4034 Optimum, Cat# 04034, Stem Cell Technologies). 10^3^ CD34^+^ cells were plated per 35 mm dish and cultured in the assay media. The number of CFU‐GM, BFU‐E, and total colonies (>100 cells) was quantified 14 d after plating using a Nikon light microscope. Each sample was run in duplicates.

### 4.4 Animals

Eight‐week‐old female NOD.Cg‐Prkdc^scid^Il2rg^tm1Wjl^/SzJ (NSG mice; Jax Lab Stock # 005557) were used in this study. The Animal Care Committee of the University Health Network approved all experimental procedures, which were carried out according to the Guide for the Care and Use of Laboratory Animals (NIH, 8th Edition, 2011). Animals were housed in a 12‐h light:12‐h dark light cycle and provided food and water ad libitum. All animals were euthanized by isoflurane overdose followed by cervical dislocation for all terminal experiments. The data presented in all figures represent one donor per mouse. Each *n*‐value represents a mouse receiving bone marrow cells from a single patient.

### 4.5 Bone marrow reconstitution

NOD‐scid‐IL2rγ^null^ mice were irradiated at 285cGy twenty‐four hours prior to injection using a Gammacell 40 Extractor Cesium‐137 Irradiator (Best Theratronics). The following day, animals received an intravenous (tail vein) injection of freshly isolated 0.7 x 10^6^ CD34^+^ or CD34^−^ cells. Cells used for all reconstitution studies were freshly isolated; cells were not frozen or expanded ex vivo. BM reconstitution analyses and myocardial infarction studies were performed 12 weeks after transplant.

### 4.6 Myocardial infarction (MI) model and functional measurements

Myocardial infarction was performed 12 weeks after bone marrow reconstitution. Each mouse was assigned a number, and the individuals conducting the operation and the functional experiments/analysis were blinded to the groups. Mice were anesthetized, intubated, and maintained with 2% isoflurane. A left thoracotomy was performed and the left anterior descending (LAD) coronary artery ligated 2 mm below the left auricle using 70 proline suture. MI was confirmed using echocardiography. Cardiac function was measured by echocardiography using a GE vivid 7i ultrasound machine equipped with the I13L probe. Animals were anesthetized, shaved, and maintained under light anesthesia (1.5% isoflurane) for all measurements. Hearts were visualized on B‐mode at the level of the papillary muscle in left ventricular long and short axis views at different time points before and after MI, 1‐, 2‐, and 4 weeks post‐ligation. Functional measurements were taken using M‐Mode at the mid‐papillary level for all mice. At study end, the akinetic wall length was measured on the B‐Mode short axis view at end diastole using cardiac echocardiography. The endocardial length of the akinetic region over the left ventricular endocardial circumference was used to measure the infarct tissue length as a percent of the left ventricle.

### 4.7 Histology and immunofluorescence

At 4 weeks or 3 days post‐MI, animals were euthanized, hearts and spleens were collected and fixed in 10% neutral buffered formalin (NBF) for 24 h for morphometry analyses or fixed in 2% paraformaldehyde (PFA) for 24 h for immunofluorescence analyses. NBF fixed hearts were processed, paraffin embedded, 5 µm sections collected and stained with Masson's trichrome. Infarct expansion index was quantified as the average of the %endocardium and %epicardium scar length relative to the respective LV lengths. To determine cardiomyocyte cross‐sectional area, Alexa 555 conjugated wheat germ agglutinin (WGA, Invitrogen, Cat#: W32464) staining was performed. Sections were stained with WGA (1:100) for 1 h at room temperature. Cross‐sectional area was measured from >100 myocytes in 3 distinct field of views within the remote region. Following fixation with PFA hearts and spleens were placed in 10%, 20%, and 30% sucrose in phosphate‐buffered saline (PBS) for 24 h at 4°C per solution for cryoprotection. After fixation, the tissues were embedded in optical cutting temperature (OCT) and 5‐8 μm sections were collected. For immunofluorescence staining, the slides were stained using the Mouse on Mouse (M.O.M) Immunodetection Basic Kit (Vector Laboratories, BMK‐2202) according to the manufacturer's instructions. Briefly, tissue sections were blocked with M.O.M. Mouse IgG Blocking Reagent for 1 h, followed by incubation in primary antibodies (Table S2) diluted in M.O.M. Diluent. After washing the slides with PBS, the samples were incubated with respective secondary antibodies (Goat anti‐Rat 488 (Invitrogen, 11–4015–82), Goat anti‐Rabbit 647 (Invitrogen, A32733), Goat Anti‐Mouse (Thermo Fisher, A‐11001), or Goat anti‐Rat (Thermo Fisher, A11081)) at room temperature in the dark for 1 h. Nuclei were stained using a 0.05% 4′,6‐diamidino‐2‐phenylindole (DAPI) solution. Quantification was carried out on heart sections scanned with the VS120 XM10 Slide Scanner (Olympus, BX61VS) at 20x magnification using either the FITC, TRITC, or CY5 filter sets. For cell counting, two random regions (0.3 mm^2^) were selected from the infarct or peri‐infarct areas from each group. Quantification was carried out on 8 µm spleen sections scanned with the VS120 XM10 Slide Scanner (Olympus, BX61VS) at 10x magnification using either the FITC, TRITC, or CY5 filter sets. For counting, cells were averaged across three randomly selected regions (1.0 mm^2^). Positively labeled cells were individually counted using Image J. For isolectin staining, and quantification was performed by quantifying fluorescence intensity (mean gray value) using ImageJ.

### 4.8 Flow cytometry

The BM, blood, spleen, and heart were collected from mice 12 weeks post‐reconstitution for baseline analyses as well as 3 and 7 day post‐MI for infarction analyses. The BM, blood, and spleen cells were isolated, and red blood cells removed through red blood cell lysis. Hearts were minced and digested using collagenase type II (Worthington, 2 mg/ml) at 37°C for 30 min and filtered through a 70 µm filter. Single cell suspensions were re‐suspended in FACS Buffer (PBS Ca2^+^/Mg2^+^ free +1% FBS). Cells were blocked with anti‐mouse CD16/32 (1:100, Biolegend) and Human Fc block (1:100, BD Biosciences) for 15 min on ice, after which cells were stained with the corresponding primary antibodies (Table S2). All primary antibody incubations were carried out for 1 h at 4°C in the dark after which cells were washed and events collected on a LSRII flow cytometer equipped with a violet laser (25 mW), blue laser (50 mW), yellow laser (100 mW), and red laser (40 mW). For identification of mouse cells, monocytes were CD45^+^/CD11b^+^/F4/80^−^/Ly6G‐/Ly6C^hi/neg^, neutrophils CD45^+^/CD11b^+^/F4/80^−^/Ly6G^+^, and macrophages CD45^+^/CD11b^+^/F4/80^+^. Gating was determined by FMOs, and all data were analyzed in FlowJo (TreeStar). For experiments using patient mononuclear cells, the isolated cells were stained for cell surface markers as described above, after which cells were analyzed on a LSR II flow cytometer. Gating used is shown in Figures S1, S2, and S7.

### 4.9 Gelatin zymography

Infarcted hearts were homogenized in liquid nitrogen using mortar and pestle and kept on ice for an hour in zymography lysis buffer (5% glycerol, 0.1% TritonX‐100 in 120 mM Tris buffer (pH 8.7)). After centrifugation (10,000 *g*), the supernatant was assayed for protein concentration using the DC protein assay (Bio‐rad) according to the manufacturer's instructions. Twenty microgram of protein was electrophoresed in an 8% gelatin gel under non‐reducing conditions. After electrophoresis, the gel was washed in 2.5% Triton X‐100 for three times and incubated in developing buffer (49.1 mM Tris, 4.81 mM CaCl2, and 0.002% NaN3 at 37 °C for 24 h). The gel was stained with 0.5% Coomassie Blue R‐250 and de‐stained using 50% methanol and 10% acetic acid solution in water. The bands representing MMP9 and MMP2 were quantified using the ImageJ software.

### 4.10 Protein isolation and cytokine analysis

Heart samples homogenized in liquid nitrogen using mortar and pestle. The total protein was extracted from powdered tissue using tissue lysis buffer (5% glycerol, 0.1% TritonX‐100 in 120 mM Tris buffer [pH 8.7]). For analysis of human cytokine, abundance protein was quantified using the DC protein assay (Bio‐rad) and suspended at a concentration of 900 µg/ml in lysis buffer supplemented with 0.5% BSA. Cytokines were quantified using the MILLIPLEX MAP Human TH17 Magnetic Bead Panel for anti‐human IFNγ, IL‐10, IL‐1β, IL‐6, IL‐4, and IL‐2. All samples and standards preparation as well as data acquisition was performed by the Princess Margaret Genomics Centre core facility (University Healthy Network, Toronto, Canada). Mouse IL‐10 (Cat# 88–7105), TNFα (Cat# 88–7346), IL‐1β (Cat# 88–7013), and IL‐6 (Cat# 88–7064) levels quantified according to the manufacturer's instructions (Thermo Fisher Scientific) using 100 µg of protein lysate per well. Standard curves were fit, and samples interpolated using Prism 8.

### 4.11 Statistical analysis

All values are expressed as mean ± SEM. Analyses were performed using GraphPad Prism 8.0 software. Statistical comparisons were done using, an unpaired two‐sided Student's *t* test, one‐way, or two‐way analysis of variance followed by a Tukey's post hoc for multiple comparisons. Values of *p* ≤ 0.05 were considered statistically significant.

## CONFLICT OF INTERESTS

None.

## AUTHOR CONTRIBUTIONS

TBM, FJA, and RKL conceptualized and designed the study. TBM, FJA, AF, SHL, LW, and JW performed experiments. TBM, FJA, and LW analyzed data. RJC, MO, and TY performed patient sample collection. All authors drafted and edited the manuscript. All authors approved the final manuscript.

## Supporting information

Supplementary Figures

## Data Availability

The data that support the findings of this study are available from the corresponding author upon request.
