# Supplementary material for: Aging impairs human bone marrow function and cardiac repair following myocardial infarction in a humanized chimeric mouse
Source: Aging Cell. 2021 Oct 6;20(11):e13494. doi: 10.1111/acel.13494 (PMC8590094; doi:10.1111/acel.13494)
Supplement: Supplementary file 1 — Supplementary Figures [file ACEL-20-e13494-s001.pdf]

Supplementary Table 1. Echocardiography analysis of cardiac function post-MI

	**Y-CD34⁺**	**O-CD34⁺**	**CD34⁻**
**Baseline**	n=7	n=7	n=10
LVIDd (mm)	3.65±0.06	3.60±0.01	3.63±0.04
LVIDs (mm)	2.19±0.04	2.16±0.01	2.17±0.03
EF (%)	77.22±0.18	77.03±0.27	77.5±0.17
FS (%)	40.18±0.18	40.08±0.24	40.47±0.18
HR (bpm)	474.23±15.96	457.13±14.45	474.61±12.28
**1 week post-MI**			
LVIDd (mm)	3.68±0.02	3.81±0.05	3.89±0.05
LVIDs (mm)	2.49±0.03	2.61±0.03	2.73±0.05*
EF (%)	67.58±0.90	65.88±0.38	63.53±1.35
FS (%)	32.29±0.67	31.22±0.35	29.72±0.93
HR (bpm)	461.68±10.66	467.51±14.31	490.49±14.71
**2 weeks post-MI**			
LVIDd (mm)	3.77±0.04**	3.88±0.04	4.01±0.06
LVIDs (mm)	2.52±0.04*	2.73±0.03	2.88±0.06
EF (%)	68.67±1.06*	63.81±1.09	61.26±1.01
FS (%)	33.30±0.77*	29.78±0.61	28.14±0.62
HR (bpm)	442.34±22.40	443.37±9.55	475.14±12.43
**4 weeks post-MI**			
LVIDd (mm)	3.79±0.06*	3.98±0.03	4.09±0.05
LVIDs (mm)	2.56±0.07*	2.85±0.04†	3.05±0.06
EF (%)	67.78±1.29*	62.99±1.13†	57.22±1.05
FS (%)	32.61±0.93*	29.34±0.74†	25.65±0.62
HR (bpm)	450.51±16.45	453.27±24.63	474.21±12.92
Infarct length (%LV)	23.49±0.64*	28.56±0.97†	37.63±2.54

LVIDd: left ventricle (LV) internal dimensions at diastole, LVIDs: LV internal dimensions at systole, EF: % ejection fraction, FS: % fractional shortening, HR heart rate. Values are mean±SEM, *p < 0.05 Y-CD34+ vs. all groups at same time point, †p<0.05 O-CD34+ vs. CD34- at same timepoint, and **p<0.05 Y-CD34+ vs. CD34- at same time point.

Supplementary Table 2. Antibody List

Antibody	*Clone*	*Product#*	*Company*
Flow Cytometry: Anti-Human Antibodies			
CD34	4H11	12-0349-42	Invitrogen
CD90	5E10	555596	BD Biosciences
CD33	WM53	303403	Biolegend
CD13	WM15	301703	Biolegend
CD45	HI30	555485	BD Biosciences
CD3	HIT3a	561802	BD Biosciences
CD19	HIB19	562441	BD Biosciences
CD14	M5E2	561385	BD Biosciences
CD45	2D1	368515	Biolegend
CD4	A161A1	357407	Biolegend
CD38	HIT2	303515	Biolegend
CD45R	HI100	304121	Biolegend
CD11b	M1-70	101206	Biolegend
Lineage Cocktail	UCHT1	348801	Biolegend
Flow Cytometry: Anti-Mouse Antibodies			
CD45	30-F11	103128	Biolegend
CD11b	M1-70	101208	Biolegend
Ly6C	HK1.4	128026	Biolegend
Ly6G	1A8	127614	Biolegend
F4/80	BM8	123114	Biolegend
CD206	C068C2	141704	Biolegend
Immunohistochemistry			
Anti-Human CD45	HI30	555480	BD Biosciences
Isolectin GS-IB4 AF568	N/A	121412	Invitrogen
Anti-Human Ku80	C48E7	2180	Cell Signaling
Wheat Germ Agglutinin	N/A	W32464	Invitrogen
Anti-Mouse CD31	MEC 13.3	550274	BD Biosciences
Anti-Mouse CD45	30-F11	550539	BD Biosciences
Anti-Muman CD31	WM59	303101	Biolegend
Anti-Ki67	SP6	ab16667	Abcam

Supplementary Figure Legends

Supplementary Figure 1. Characterizing patient blood and bone marrow hematopoietic lineage. a) Patients' complete blood count measure prior to surgery quantifying the total white blood cell, neutrophil, monocytes, and lymphocytes in circulation, mainly driven by increased neutrophils and monocytes (n=39 young and n=34 old). b) Bone marrow cellularity, as measured by mononuclear cells correlated with patient age (n=88). c) Frequency of $CD34^+$ cells grouped by age group (n=23 young and n=28 old) and also d) correlated with patient age (n=51). e) Representative flow cytometry gating used to quantify the hematopoietic progenitor population. f) Frequency of patient BM lineage progenitor populations measured by flow cytometry based on multipotent progenitor cells (MPP), multi-lymphoid progenitors (MLP), g) hematopoietic stem cell frequency (HSC) (n=4 young and n=7 old) and h) early versus committed progenitors between the young and old patient cohort (committed: n=8 young and n=10 old). Values are mean±SEM. *p<0.05 vs. indicated groups.

Supplementary Figure 2. a) Gating used to identify neutrophil and monocyte populations in patient bone marrow and b) quantification. c) Representative flow cytometry gating of human and mouse CD45 cells in the BM of NSG mice 3 months after reconstitution (left) and quantification (right). Values are mean±SEM. *p<0.05 vs. indicated groups.

Supplementary Figure 3. CD34 purity and immune cell mobilization at baseline post-reconstitution. a) Representative flow cytometry data demonstrating purity of CD34 cell isolation after magnetic activated cell sorting. b) Human $CD45^+$ cell frequency

in blood and spleen of Y/O $CD34^+$ and $CD34^-$ myocardium 3 months after reconstitution n=14-18/group. c) Quantification of akinetic wall region as % of LV at 4 weeks post-MI by echocardiography (n=7-10/group). d) Staining of human CD45 and the pan human nuclear marker Ku80 in the infarct region of Y-CD34+ and O-CD34+ hearts at 3d post-MI (top) and staining of human CD31 and the pan human nuclear marker Ku80 in the infarct region at 4w post-MI (bottom). *p<0.05 vs. all other groups, and †p<0.05 vs. CD34-. Values are mean±SEM.

Supplementary Figure 4. Cardiac function post-MI in reconstituted and WT NSG mice. a) LV dimensions at diastole (left) and systole (right) at baseline, 1w, 2w and 4w post-MI. b) Cardiac function as measured by ejection fraction (left) and fractional shortening (right) at baseline, 1w, 2w and 4w post-MI (n=7-10/group). c) Representative gross tissue sections showing scar size in Y-CD34+, O-CD34+, CD34-, and WT NSG mice 4 weeks post-MI. *p<0.05 Y-CD34+vs. all other groups, and **p<0.05 Y-CD34+ vs. CD34- and WT NSG, †p<0.05 O-CD34+ vs. CD34-, ‡p<0.05 Y-CD34+ vs. WT NSG by two-way ANOVA followed by Tukey post-hoc. Values are mean±SEM.

Supplementary Figure 5. B-cell analysis in myocardium, blood and spleen at baseline and post-MI. B-cell frequency in Y/O $CD34^+$ myocardium at baseline, 3- and 7-days post-MI in a) myocardium (n=4-10/group) and b) blood (n=7-10/group). c) Total immune cells in spleen of Y/O $CD34^+$ reconstituted mice were quantified using $hCD45^+$ at baseline, 3- and 7-days post-MI (n=7-10/group). d) B-cell frequency in the spleen of these animals were also quantified at these time points (n=7-10/group). e) Representative

dot plots of human CD3+/CD4+ cells in the heart following infarction. Values are mean±SEM.

Supplementary Figure 6. a) Representative staining of mouse CD45+ cells within the infarct region in Y-CD34+, O-CD34+, CD34-, and WT NSG hearts at 3 post-MI (left) and higher magnification images within the infarcted myocardium (right). b) Quantification of mouse CD45+ (mCD45) cells in the infarct and peri-infarct regions. *p<0.05 vs. WT NSG and †p<0.05 vs. CD34-, by one way ANOVA followed by Tukey post-hoc. All values are mean±SEM

Supplementary Figure 7. a) Representative gating used to identify human cells in the infarcted myocardium as well as FMOs used to determine gates. b) Representative dot plots of mouse monocytes and neutrophils in the infarcted myocardium 3 days post-MI

Supplementary Figure 8. a) Representative images used for quantification of human CD45 and Ki67 cells in the spleen at 3d post-MI. Quantification of b) human CD45 cells, c) hCD45+/Ki67+, and d) hCD45-/Ki67+. *p<0.05 vs. all other groups and †p<0.05 vs. O-CD34+. All values are mean±SEM

Marvasti et al. – Supplementary Figure 1

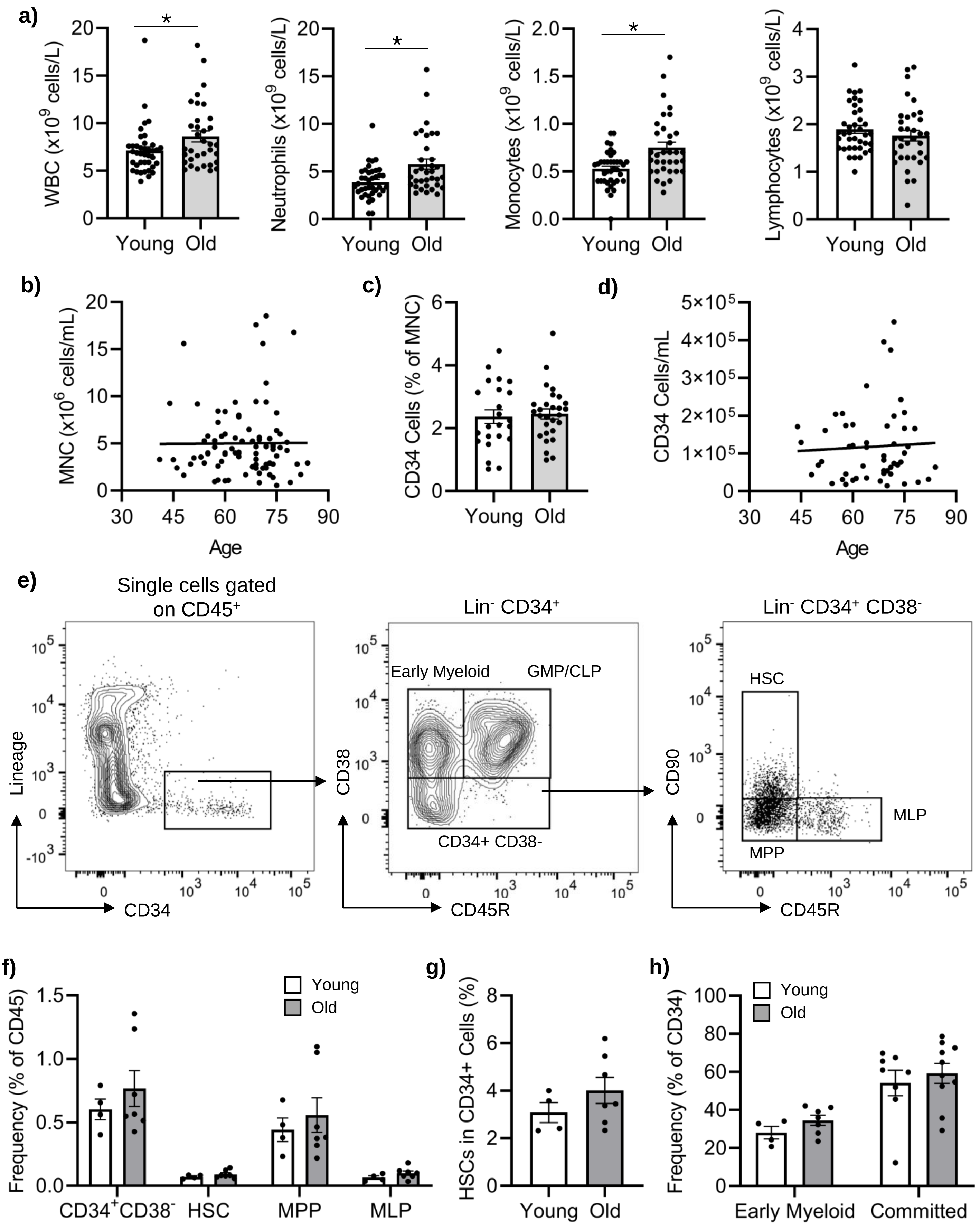

Marvasti et al. – Supplementary Figure 2

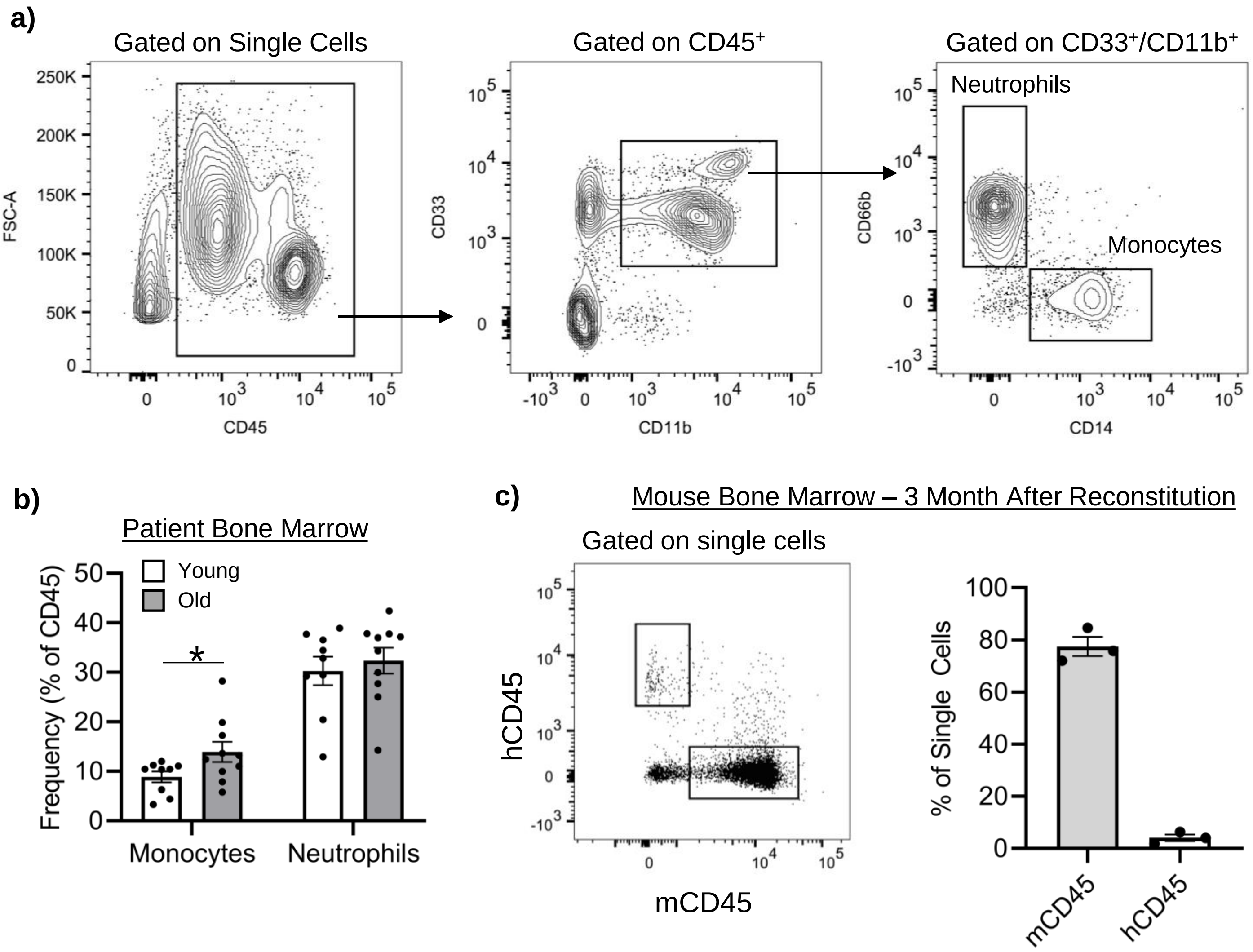

Marvasti et al. – Supplementary Figure 3

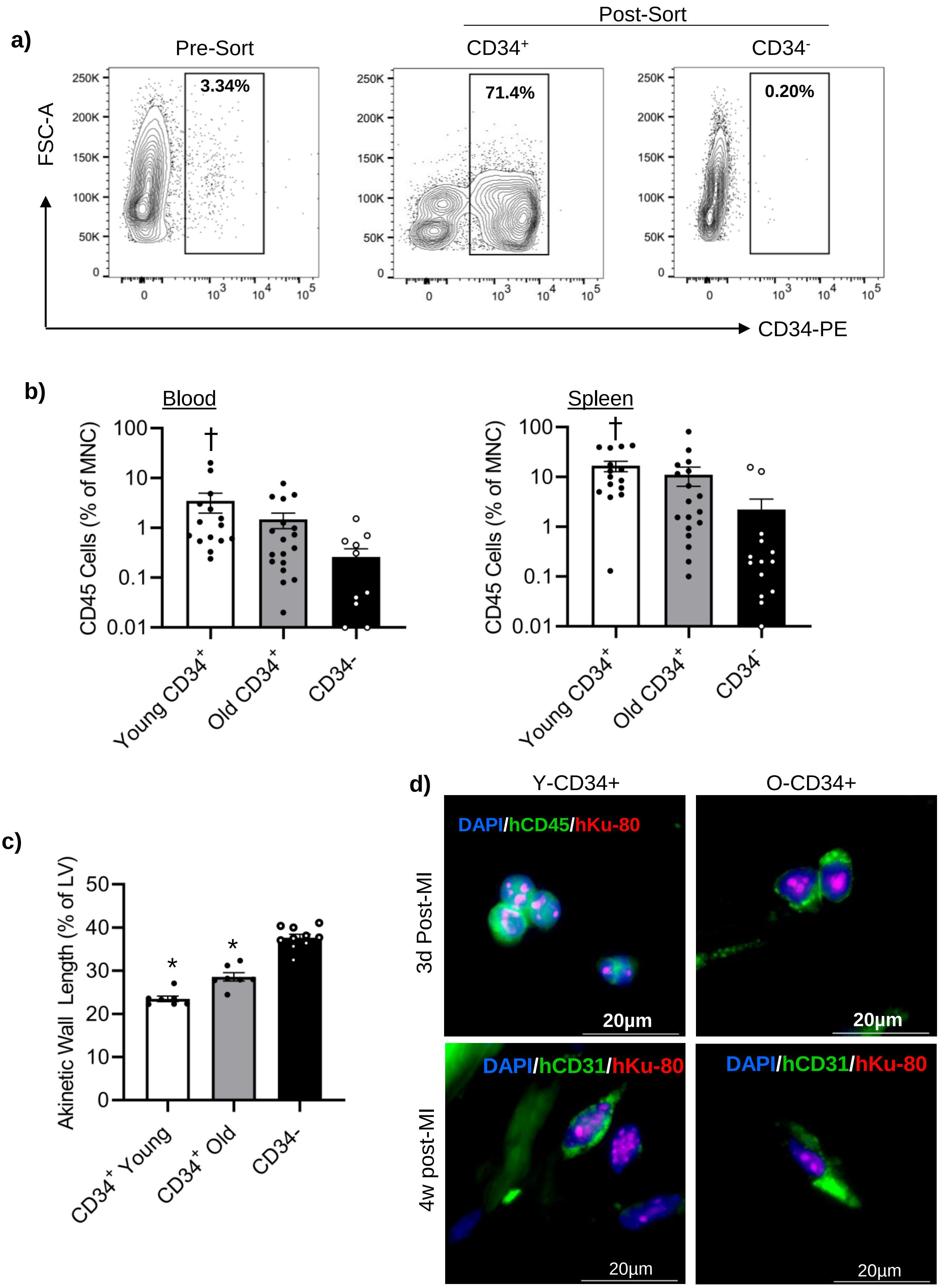

Marvasti et al. – Supplementary Figure 4

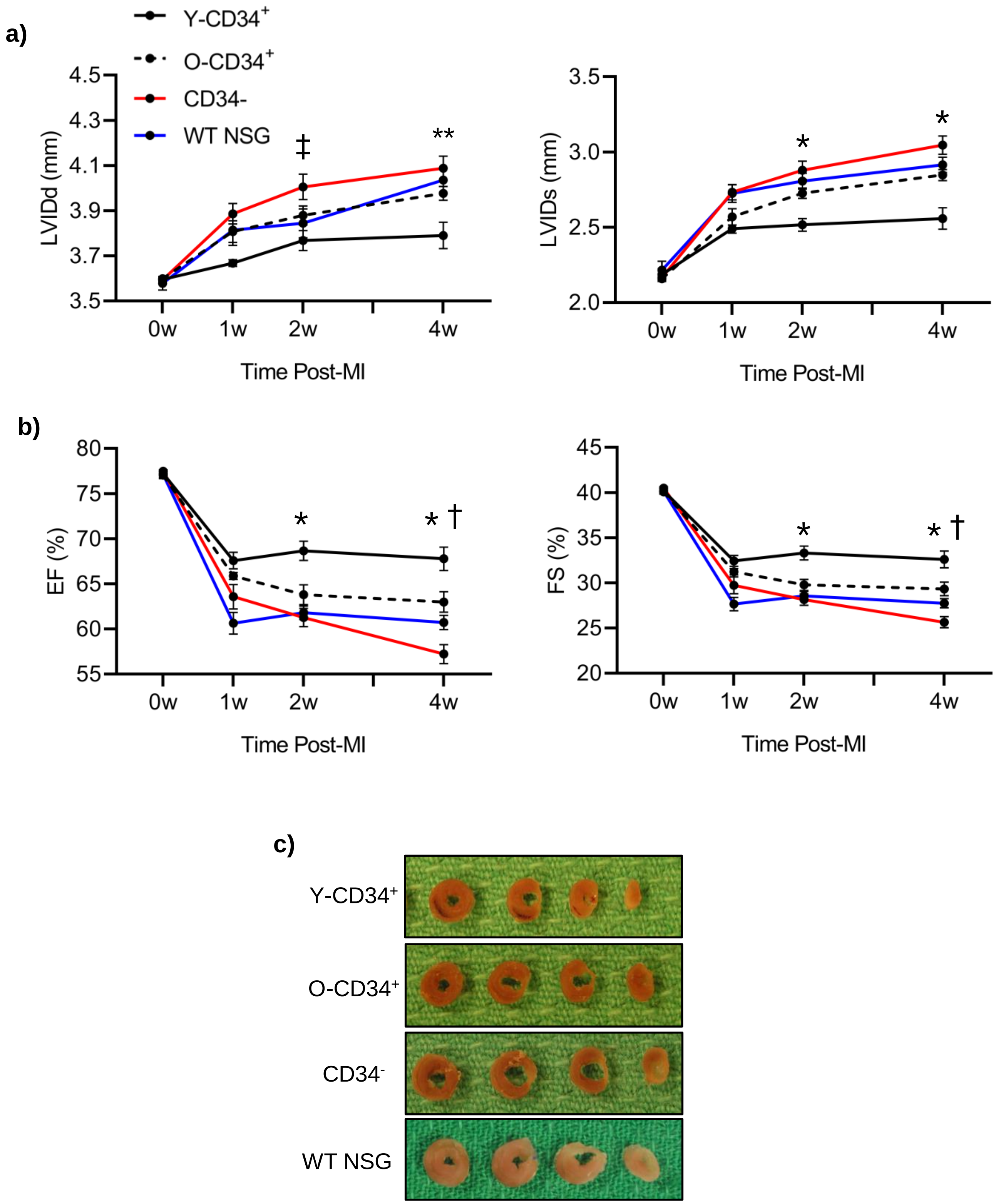

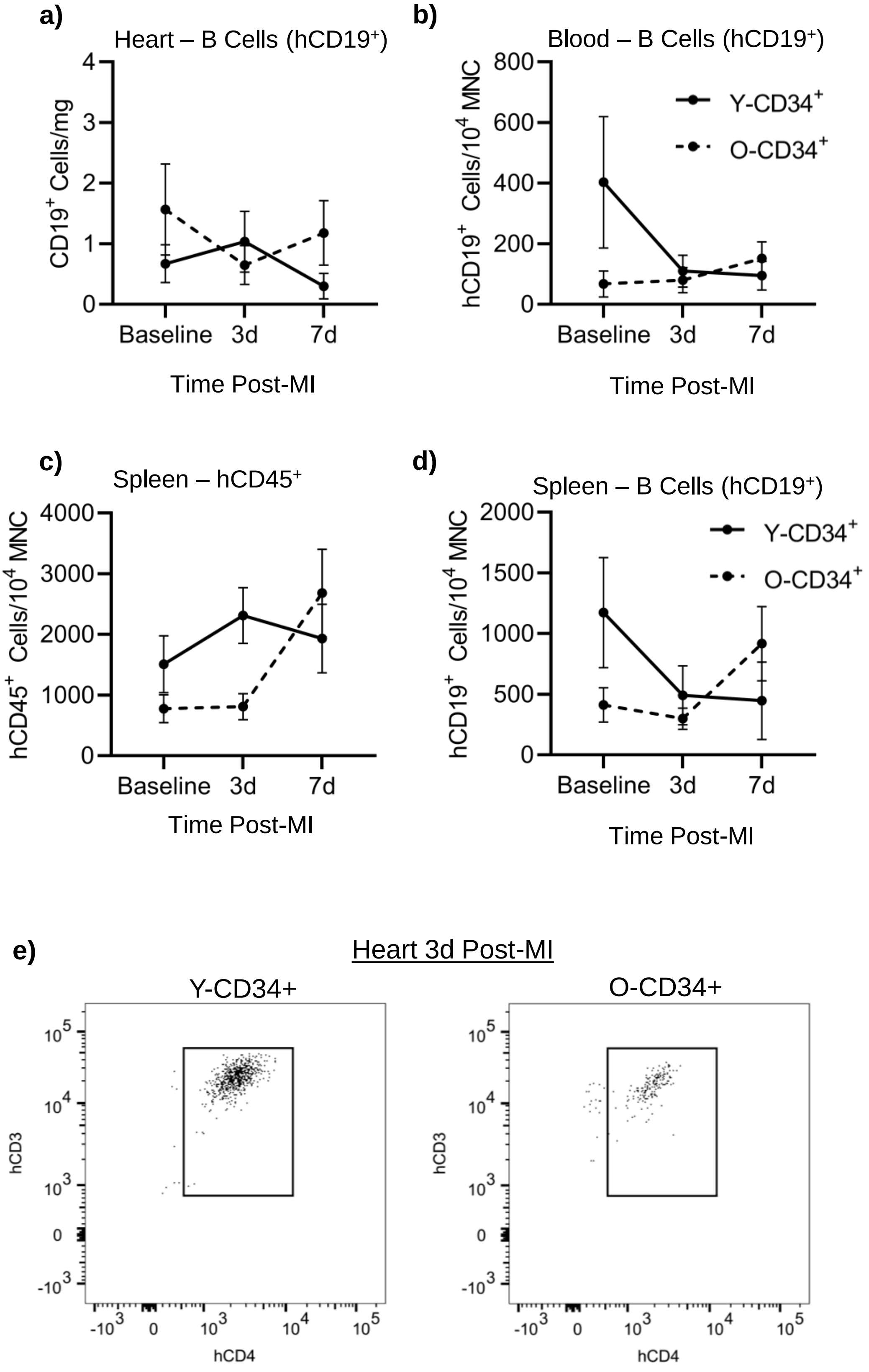
a)
Heart – B Cells (hCD19+)
CD19+ Cells/mg
Baseline
3d
7d
Time Post-MI
b)
Blood – B Cells (hCD19+)
hCD19+ Cells/10^4 MNC
Y-CD34+
O-CD34+
Time Post-MI
c)
Spleen – hCD45+
hCD45+ Cells/10^4 MNC
Time Post-MI
d)
Spleen – B Cells (hCD19+)
hCD19+ Cells/10^4 MNC
Y-CD34+
O-CD34+
Time Post-MI
e)
Heart 3d Post-MI
Y-CD34+
O-CD34+
hCD3
hCD4

Marvasti et al. – Supplementary Figure 6

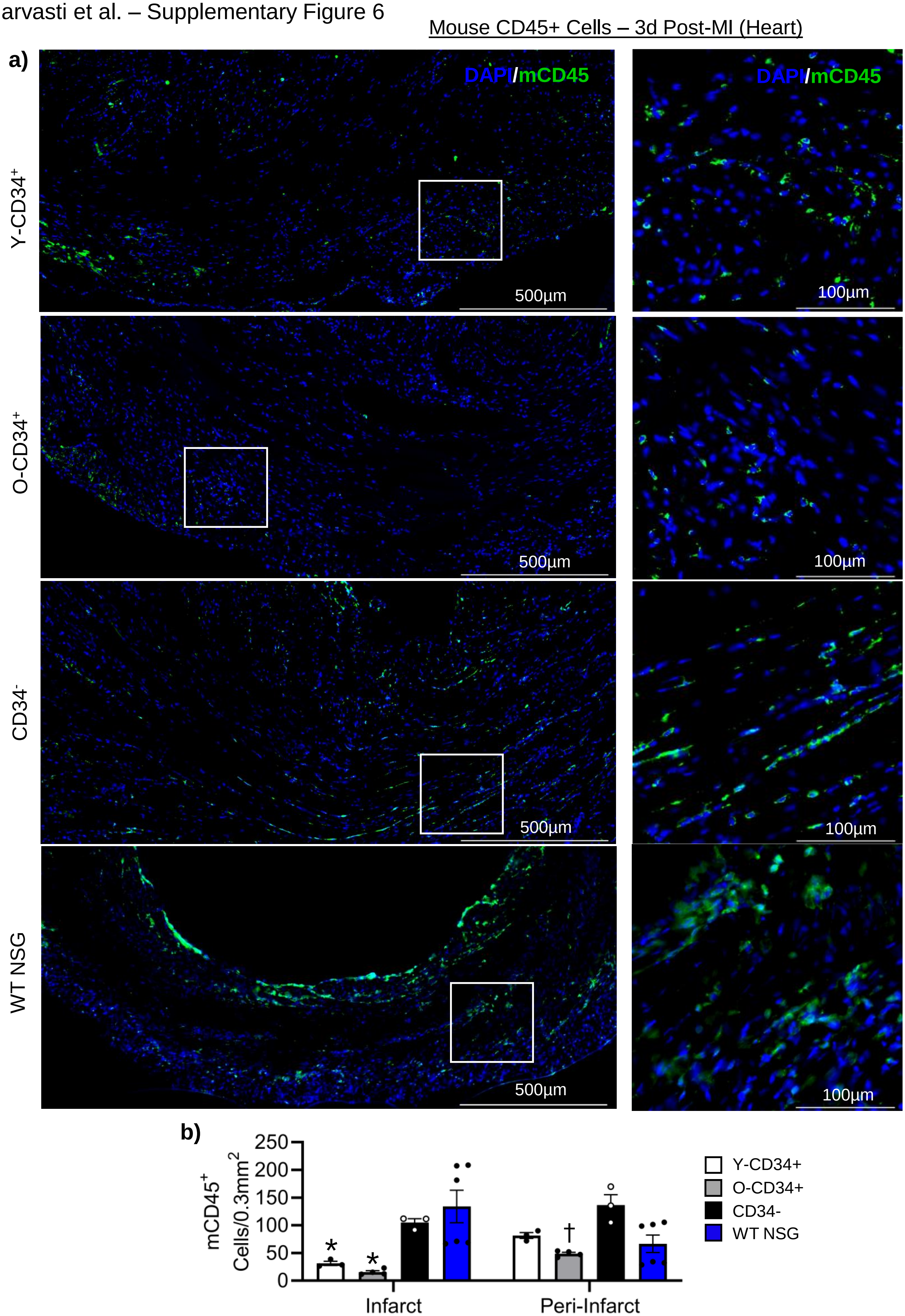

Marvasti et al. – Supplementary Figure 7

a) Representative Gating for Identification of Human Cells

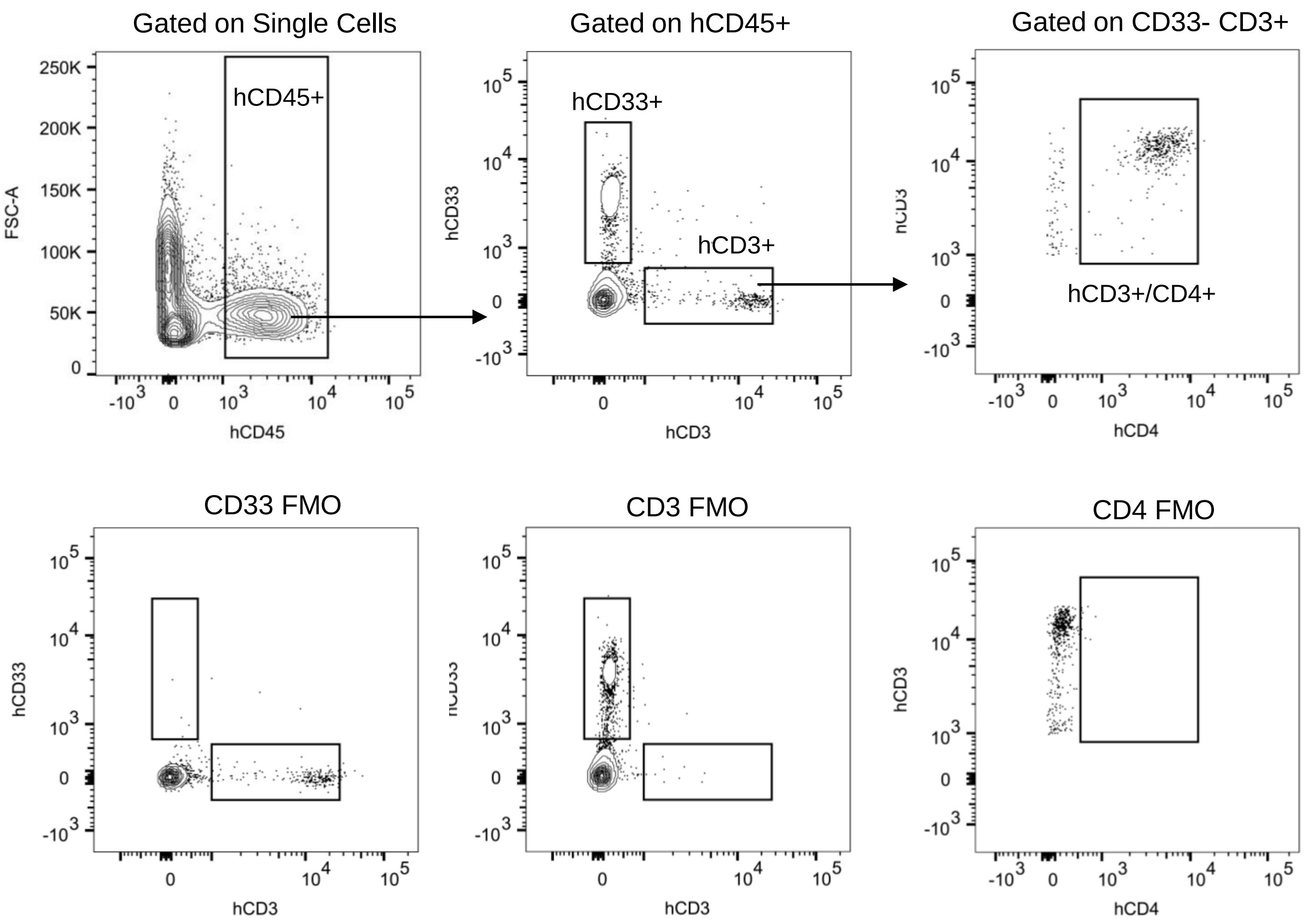

b) Representative Plots of Mouse Myeloid Cells in the Heart 3d Post-MI (Gated on CD11b+ F4/80-)

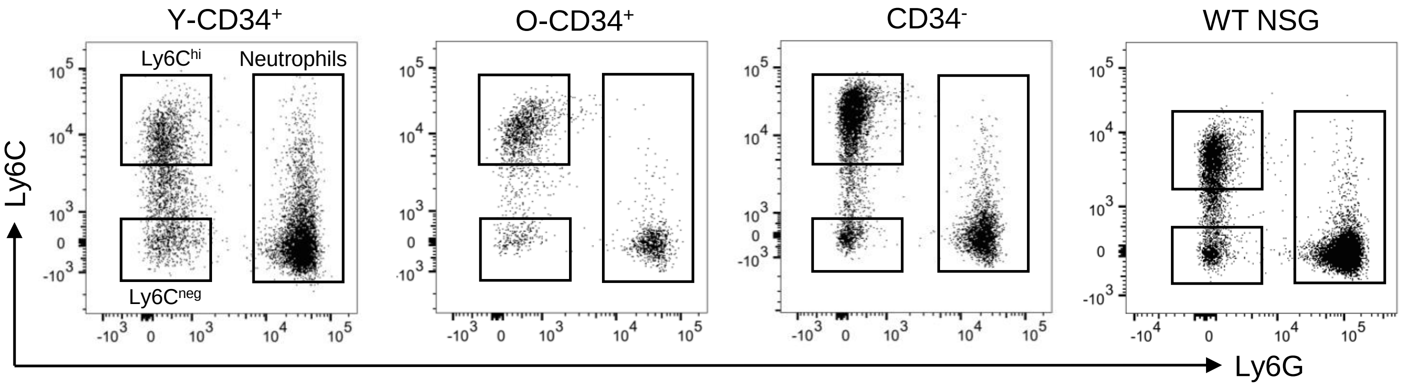

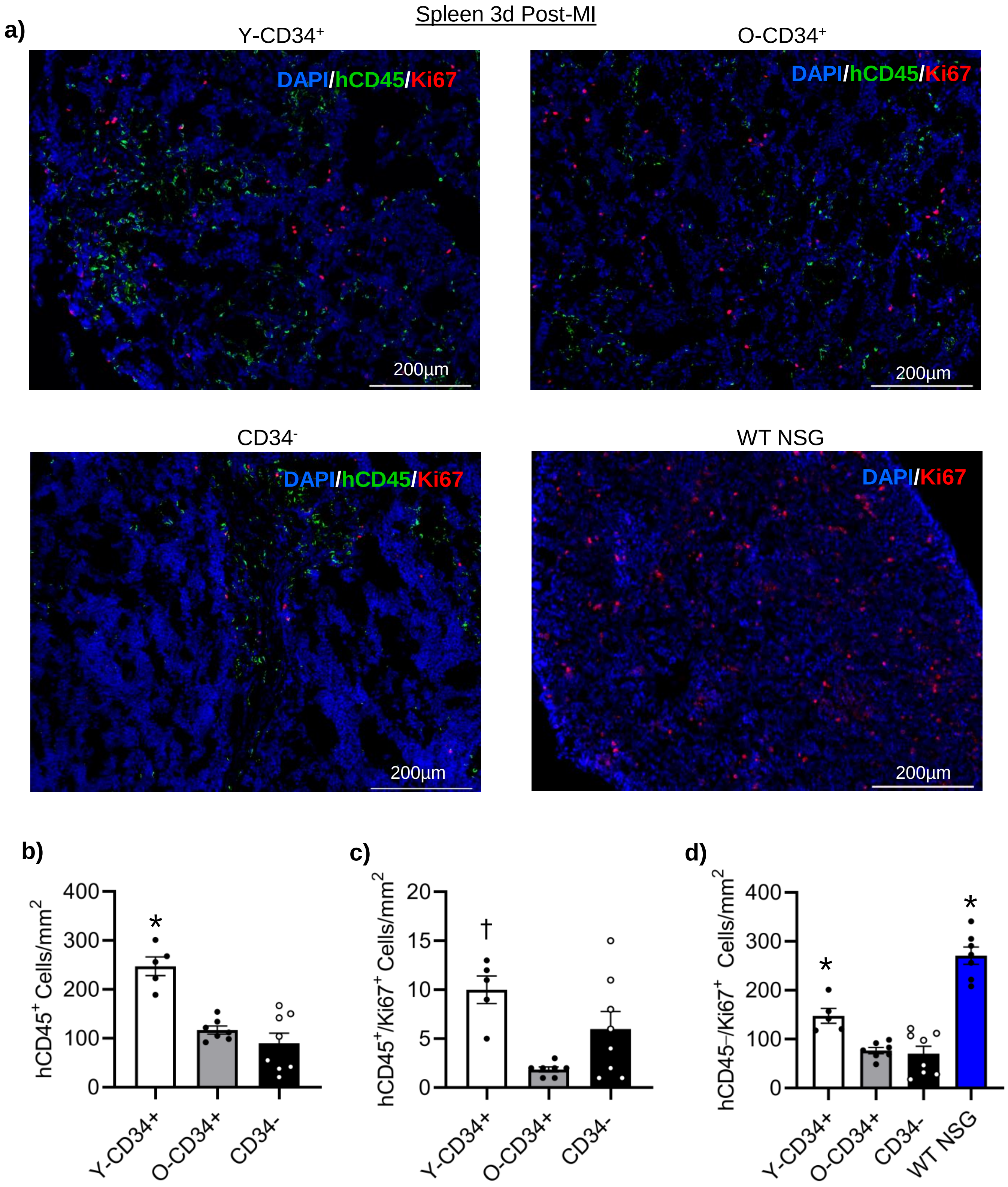
a)
Spleen 3d Post-MI
Y-CD34+
O-CD34+
CD34-
WT NSG
DAPI/hCD45/Ki67
DAPI/Ki67
200µm
b)
hCD45+ Cells/mm2
400
300
200
100
0
Y-CD34+
O-CD34+
CD34-
*
c)
hCD45+/Ki67+ Cells/mm2
20
15
10
5
0
†
d)
hCD45–/Ki67+ Cells/mm2
WT NSG
